# Impaired Chromatin Remodelling at STAT1-Regulated Promoters Leads to Global Unresponsiveness of *Toxoplasma gondii*-Infected Macrophages to IFN-γ

**DOI:** 10.1371/journal.ppat.1002483

**Published:** 2012-01-19

**Authors:** Christine Lang, Anke Hildebrandt, Franziska Brand, Lennart Opitz, Hassan Dihazi, Carsten G. K. Lüder

**Affiliations:** 1 Institute for Medical Microbiology, University Medical Center, Georg-August-University, Göttingen, Germany; 2 DNA Microarray Facility, University Medical Center, Georg-August-University, Göttingen, Germany; 3 Department of Nephrology and Rheumatology, University Medical Center, Georg-August-University, Göttingen, Germany; UMDNJ-New Jersey Medical School, United States of America

## Abstract

Intracellular pathogens including the apicomplexan and opportunistic parasite *Toxoplasma gondii* profoundly modify their host cells in order to establish infection. We have shown previously that intracellular *T. gondii* inhibit up-regulation of regulatory and effector functions in murine macrophages (MΦ) stimulated with interferon (IFN)-γ, which is the cytokine crucial for controlling the parasites' replication. Using genome-wide transcriptome analysis we show herein that infection with *T. gondii* leads to global unresponsiveness of murine macrophages to IFN-γ. More than 61% and 89% of the transcripts, which were induced or repressed by IFN-γ in non-infected MΦ, respectively, were not altered after stimulation of *T. gondii*-infected cells with IFN-γ. These genes are involved in a variety of biological processes, which are mostly but not exclusively related to immune responses. Analyses of the underlying mechanisms revealed that IFN-γ-triggered nuclear translocation of STAT1 still occurred in *Toxoplasma*-infected MΦ. However, STAT1 bound aberrantly to oligonucleotides containing the IFN-γ-responsive gamma-activated site (GAS) consensus sequence. Conversely, IFN-γ did not induce formation of active GAS-STAT1 complexes in nuclear extracts from infected MΦ. Mass spectrometry of protein complexes bound to GAS oligonucleotides showed that *T. gondii*-infected MΦ are unable to recruit non-muscle actin to IFN-γ-responsive DNA sequences, which appeared to be independent of stimulation with IFN-γ and of STAT1 binding. IFN-γ-induced recruitment of BRG-1 and acetylation of core histones at the IFN-γ-regulated CIITA promoter IV, but not β-actin was diminished by >90% in *Toxoplasma*-infected MΦ as compared to non-infected control cells. Remarkably, treatment with histone deacetylase inhibitors restored the ability of infected macrophages to express the IFN-γ regulated genes *H2-A/E* and *CIITA*. Taken together, these results indicate that *Toxoplasma*-infected MΦ are unable to respond to IFN-γ due to disturbed chromatin remodelling, but can be rescued using histone deacetylase inhibitors.

## Author Summary

*Toxoplasma gondii* is a common unicellular parasite of humans and other vertebrates and can lead to overt disease mostly in immune-suppressed patients or in fetuses. Since IFN-γ is the major mediator of resistance against *T. gondii*, inhibition of IFN-γ-mediated gene expression may be a crucial mechanism to allow parasite survival in the immune-competent hosts. Here, we used genome-wide expression profiling to show that parasite infection renders murine macrophages globally unresponsive to stimulation with IFN-γ. This results in severe defects of infected macrophages to regulate a variety of immune-related, but also immune-unrelated biological pathways. By analysing the underlying mechanisms, we provide substantial evidence that *Toxoplasma* interferes with the assembly of chromatin remodelling complexes at IFN-γ-responsive DNA sequences. Furthermore, binding of the transcription factor signal transducer and activator of transcription 1 (STAT1) to IFN-γ-regulated promoters, but not its nuclear import is disturbed in infected cells. The acetylation of histones at IFN-γ-regulated promoters was found to be severely impaired. Importantly, treatment with histone deacetylase inhibitors rescues *Toxoplasma*-infected macrophages from the inability to respond to IFN-γ. Our study reveals new insights into the evasion of IFN-γ-mediated host immunity by *T. gondii*, and opens the possibility of a novel intervention strategy against *T. gondii* by modulating this parasite-host interaction.

## Introduction

*Toxoplasma gondii* is a protozoan parasite that is highly prevalent in humans and warm-blooded animals throughout the world. As a member of the Apicomplexa, it is obligatory intracellular and actively invades a broad range of both immune and non-immune cells within its host. When replicating in an uncontrolled manner, *Toxoplasma* infection can lead to severe tissue damage and life-threatening disease, as observed after transmission to fetuses in utero or after reactivation of persistent infection in immunocompromized patients [1]. In contrast, when the parasite is effectively controlled by a mature immune system, infection is mostly asymptomatic or benign but leads to *Toxoplasma* persistence for the hosts' life. The remarkable ability of the parasite to establish chronic infection in immunocompetent hosts may depend on various immune evasion strategies, which are facilitated by extensive modifications of host cells following infection [2].

Interferon (IFN)-γ is the most important cytokine that controls *T. gondii* replication *in vitro* and *in vivo* [3]–[8]. IFN-γ is highly pleiotropic and regulates more than 200 known genes, although the actual number is probably much higher [9]–[10]. During toxoplasmosis, IFN-γ is readily secreted by CD4^+^, CD8^+^ T cells and natural killer (NK) cells leading to increased serum levels in both mice and humans [5]–[6], [11]–[14]. However, we and others have previously shown that intracellular *Toxoplasma* parasites inhibit expression of several IFN-γ-regulated genes including those encoding the major histocompatibility complex (MHC) class II, inducible nitric oxide synthase (iNOS), p47 GTPases and monokine induced by gamma interferon (MIG) in macrophages (MΦ) [15]–[20]. IFN-γ-regulated gene expression is also impaired in other cell types infected with *Toxoplasma* such as astrocytes and microglia [21] as well as human fibroblasts [22]. However, interference with MΦ function may be particularly relevant, because these cells are critical for the course of infection. It has been shown that human monocytes are more readily infected, and are more permissive for parasite replication *in vitro* than other blood leukocytes [23]. Furthermore, mouse monocytes support substantial *Toxoplasma* propagation *in vivo,* and together with dendritic cells are important vehicles, which facilitate hematogenous dissemination within the host [24]–[25]. MΦ also orchestrate the immune response to *T. gondii* following infection. Inflammatory monocytes migrate into parasitized tissue, and fulfil vital antimicrobial functions that control infection at initial sites of parasite inoculation [26]–[28]. MΦ may also be required for the development of efficient Th1-type adaptive T cell responses to *T. gondii* infection [6], [29]. Recently, Lykens and colleagues established that CD68^+^ cells of the macrophage lineage and their responsiveness to IFN-γ are indeed decisive for parasite control and host mortality during toxoplasmosis [30].

IFN-γ activates gene expression mainly via the JAK/STAT1 signalling pathway resulting in the translocation of active STAT1 homodimers into the nucleus [31]. These then bind to gamma-activated sites (GAS) in the promoters of IFN-γ-responsive genes. We and others found no defects in IFN-γ-induced nuclear import of STAT1 in *T. gondii*-infected murine macrophages [19] or human fibroblasts [22]. Despite this, cells were unable to up-regulate IFN-γ-induced gene expression [19].

Gene transcription requires extensive remodelling of the three-dimensional structure of chromatin and the assembly of the transcriptional machinery at respective promoters. Covalent modifications of histones including acetylation, methylation, or phosphorylation at distinct residues have been recognized as critical factors for the control of gene expression [32]. They dictate the accessibility of DNA, and function as docking sites for the binding of proteins that are required for transcriptional activation or repression. Acetylation of histones is commonly associated with activation of transcription whereas their deacetylation correlates with repression [32]. Histones are mainly acetylated by histone acetyl transferases (HATs) the activity of which is counteracted by histone deacetylases (HDACs) [32]. Chromatin remodelling complexes constitute a second group of enzymes, which are involved in chromatin regulation and disrupt histone-DNA contacts in an ATP-dependent process [33]. The prototypic BAF (Brahma-related gene (BRG)/Brahma (BRM)-associated factor) complex (related to the yeast SWI-SNF complex) [33] is crucial for the expression of immune-related genes in response to external stimuli. It is also required for activation of the prototypic IFN-γ-responsive promoters of GBP2, CIITA and HLA-DR [34]–[37]. BAF complexes often cooperate with histone-modifying enzymes to regulate genes including those induced by IFN-γ [33]–[34], [38].

Given the crucial roles of MΦ and their responsiveness to IFN-γ for the defence against *T. gondii* on the one hand [30], and the parasite's ability to inhibit the expression of distinct IFN-γ-regulated genes on the other hand [15], [17], [19]–[20], we sought to determine the effect of *Toxoplasma* infection on IFN-γ responsiveness of MΦ on a global scale. Using transcriptome analyses, we show a general defect of infected murine MΦ to regulate gene expression after activation with IFN-γ. Subsequent mechanistic analyses revealed an impairment of parasite-infected MΦ to recruit components of chromatin remodelling complexes to STAT1-regulated promoters and to acetylate histones in response to IFN-γ. Furthermore, we provide evidence that treatment with HDAC inhibitors restores IFN-γ responsiveness of *Toxoplasma*-infected MΦ. We thus unravel key molecular mechanisms of this intracellular protozoan parasite to facilitate survival in host MΦ, which may be decisive for the successful establishment of infection.

## Results

### Unresponsiveness of *Toxoplasma*-infected macrophages to IFN-γ

**Figure 1:**
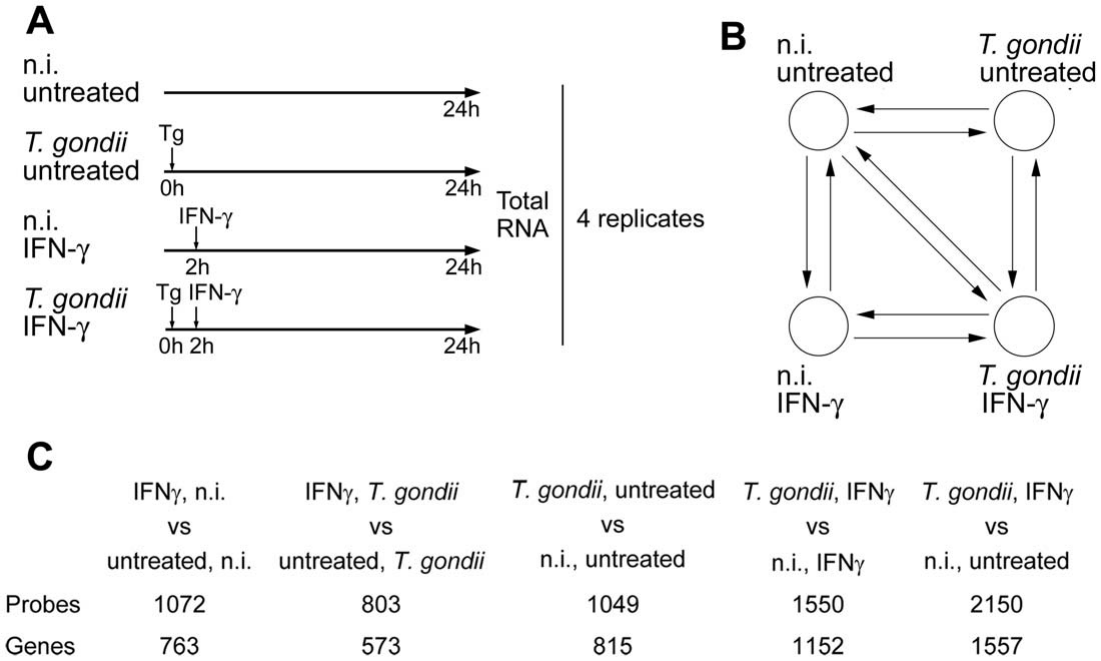
Regulation of the transcriptome of primary murine macrophages after infection with *T. gondii* and/or activation with IFN-γ. (A) Primary BMMΦ were infected or not with *T. gondii* and 2 hours later, were stimulated with IFN-γ for additional 22 hours or left untreated. RNA from four biological replicates was used for further analysis. (B) After reverse transcription of mRNA and labelling with Cy3 or Cy5, two samples each were hybridized to mouse whole genome microarrays in a dye-swap loop design as indicated by arrows. (C) Number of probes and corresponding number of genes or transcripts that were at least 4-fold regulated at ≤ 1% FDR.

In order to determine the global effect of *T. gondii* infection on IFN-γ responsiveness of macrophages, we performed whole genome microarray analyses of murine bone marrow-derived MΦ (BMMΦ), infected or not with *T. gondii*, and stimulated or not with IFN-γ (Figure 1A). Transcripts were obtained from four biological replicates each. After reverse transcription and labelling, cDNA was hybridized to microarray slides in a dye swap four sample loop design (Figure 1B) providing a good average precision and robustness [39]. Data were experimentally validated by real-time PCR. Eighteen out of twenty primer pairs specific for randomly selected genes from the microarray (Figure S1A) amplified targets of the expected size. Comparison of mRNA levels as obtained by microarray and quantitative RT-PCR revealed a regression correlation of r = 0.95, indicating a high level of concordance between both methods (Figure S1B).

Out of 41,174 good quality microarray spots, 1,072 spots representing 763 unique genes were at least 4-fold regulated (absolute change) by IFN-γ in non-infected BMMΦ at a false discovery rate (FDR) of ≤ 1% (Figure 1C; data is accessible through NCBI GEO, accession number GSE28499). In contrast, only 803 spots or 573 unique genes were regulated in *T. gondii*-infected macrophages in response to IFN-γ. This indicates that a significant number of IFN-γ-regulated genes could not be induced or repressed by BMMΦ infected with *Toxoplasma*. We also identified 815 genes that were at least 4-fold regulated in untreated macrophages after parasite infection, as compared to non-infected and untreated cells (Figure 1C). Remarkably, the number of genes regulated by *T. gondii* further increased (1152 unique genes) in IFN-γ-stimulated cells, indicating specific parasite-macrophage interactions depending on the cytokine milieu. Not surprisingly, the highest number of unique genes (1557) was found to be regulated when *Toxoplasma*-infected and IFN-γ-stimulated BMMΦ were compared with non-infected, untreated control cells (Figure 1C).

**Figure 2:**
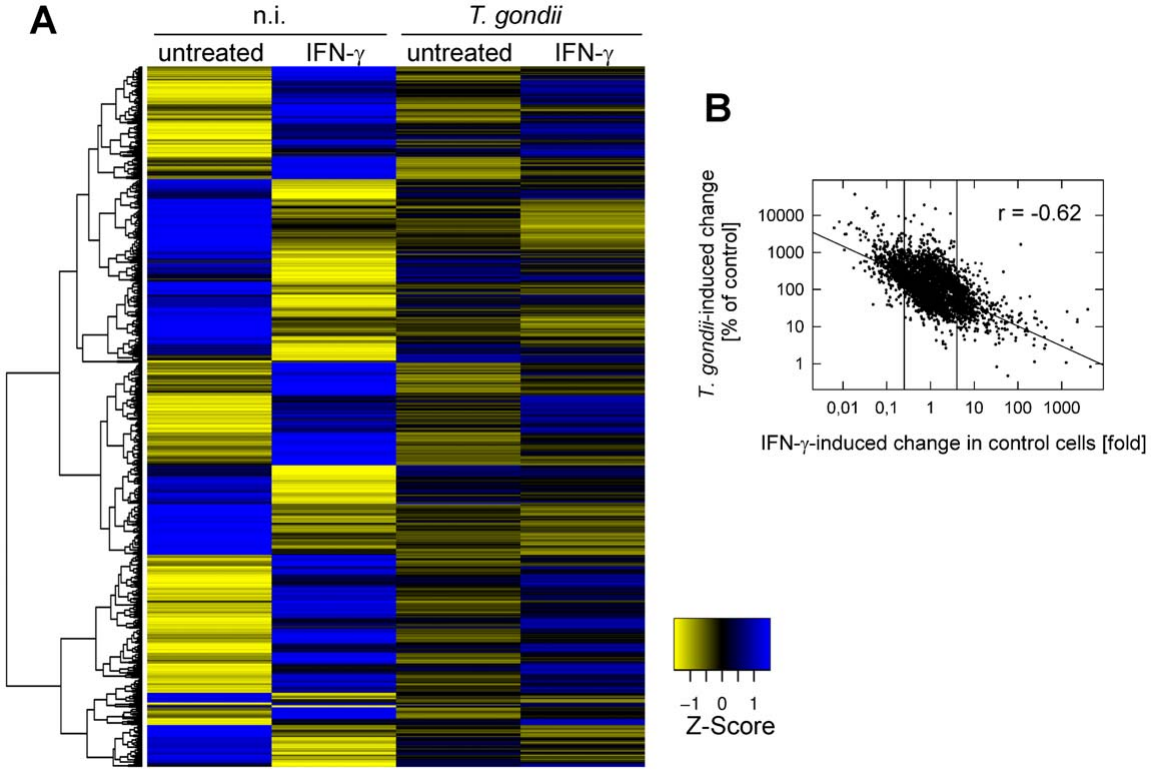
*T. gondii* infection severely impairs the ability of murine macrophages to respond to IFN-γ. (A) The cDNAs from non-infected and *T. gondii*-infected murine BMMΦ stimulated or not with IFN-γ were hybridized to mouse whole genome microarrays. Microarray probes were identified, which showed at least 4-fold up- or down-regulation in IFN-γ-treated, non-infected cells versus untreated, non-infected cells. Expression levels of the corresponding genes in non-infected (n.i.) and *T. gondii*-infected cells before and after IFN-γ treatment are displayed following Z-score transformation using a blue (induced) to yellow (repressed) scale. (B) Out of the 41,174 good quality spots, those which were differentially regulated in at least one of the five comparisons as depicted in Figure 1B were selected (threshold 4-fold regulation; identified by ANOVA; n = 3527). They were subjected to a correlation analysis of the fold change of mRNA from non-infected control cells in response to IFN-γ, and the impact exerted by *T. gondii* on IFN-γ-regulated gene expression. Vertical lines at 0.25 and 4 indicate lower and upper thresholds for differentially expressed genes. The correlation coefficient r of the linear regression is indicated.

A major goal of this study was to determine the effect of *Toxoplasma* infection on IFN-γ-triggered gene expression. Therefore, we concentrated on genes which were regulated in response to IFN-γ, comparing non-infected with *T. gondii* infected cells. After IFN-γ stimulation, 567 and 505 genes were up- or down-regulated (threshold level of ≥4-fold change), respectively, in non-infected BMMΦ (Figure 2A; for a list of genes up- or down-regulated in non-infected MΦ see Tables S1 and S2, respectively). These numbers differ strongly from those found in human-derived fibroblasts where the number of regulated genes was considerably lower (n = 127), and no genes were repressed by IFN-γ [22]. Importantly, concomitant infection with *T. gondii* largely disabled BMMΦ to respond to IFN-γ (Figure 2A). Thus, the vast majority of genes which were induced by IFN-γ in non-infected BMMΦ were not induced in *Toxoplasma*-infected cells (Figure 2A; Table S1). Among them, several genes were identified that are known to be repressed by *T. gondii*, including the MHC class II molecules *H2-Aα*, *H2-DMβ1*, *H2-Eα* [15]–[16], the interferon-regulatory factor 1 (*IRF-1*) [16], [22], the class II transactivator (*CIITA*) [16], [19], and the interferon gamma induced GTPase (*IGTP*) [18]. However, we also identified several IFN-γ-induced genes and transcripts, the expression of which was even higher in *Toxoplasma*-infected BMMΦ. These genes were up-regulated by *Toxoplasma* infection itself as observed in either non-stimulated (ratio *T. gondii*, untreated/n.i., untreated) or stimulated (ratio *T. gondii*, IFN-γ/n.i., IFN-γ) cells. This indicates that for distinct genes, parasite infection can override the overall inhibitory effect of the parasite on IFN-γ-regulated gene expression (data not shown). Contrary to IFN-γ-induced transcripts, those repressed in non-infected macrophages by IFN-γ (IFN/Ctr ≤ 0.25) were mostly increased after infection (Figure 2A; Table S2). Quantitative analysis revealed that genes, which were strongly induced or repressed by IFN-γ were also strongly inhibited or induced by *T. gondii*, respectively, whereas genes that remained unchanged after IFN-γ-treatment were also mostly unaffected by the parasite (Figure 2B). Thus, the level of IFN-γ-induced regulation negatively correlated with the change exerted by the parasite (correlation coefficient r = -0.62; *p*<0.00001).

**Figure 3:**
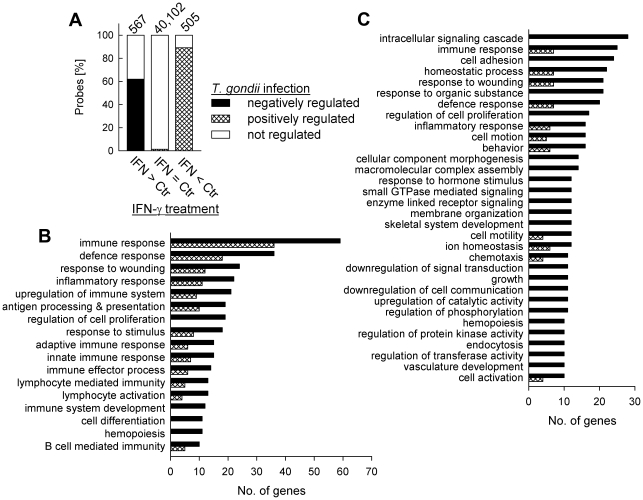
Impact of *Toxoplasma* infection on the IFN-γ-regulated transcriptome of murine BMMΦ as determined by mouse cDNA microarray analyses. (A) Oligonucleotide probes on the array that were at least 4-fold up-regulated (IFN > Ctr) or 4-fold down-regulated (IFN < Ctr) after IFN-γ treatment of non-infected BMMΦ, as compared to untreated controls were identified at a FDR ≤ 1%, and were compared with un-regulated probes (IFN  =  Ctr). Total numbers of probes are given at the top of each bar. The percentages of probes of which the up- or down-regulation in non-infected cells was abrogated in *Toxoplasma*-infected cells (infected, IFN-γ-treated vs. infected, untreated) are indicated by black and cross-hatched bars, respectively. (B) Genes that were at least 4-fold up-regulated after IFN-γ-treatment of non-infected (black bars) or *Toxoplasma*-infected BMMΦ (cross-hatched bars) were clustered in biological processes using the DAVID Bioinformatics resource. Depicted are major IFN-γ-regulated clusters containing a minimum of 10 different genes, the expression of which was at least 4-fold induced after treating non-infected macrophages with IFN-γ. (C) Genes that were at least 4-fold down-regulated by IFN-γ-treatment of non-infected (black bars) or *Toxoplasma*-infected BMMΦ (cross-hatched bars) were clustered in biological processes using the DAVID Bioinformatics resource. Depicted are major IFN-γ-regulated clusters containing a minimum of 10 different genes, the expression of which was at least 4-fold repressed after treating non-infected macrophages with IFN-γ.

More than 60% of the genes (n = 351) induced by IFN-γ in non-infected BMMΦ (based on a threshold level of ≥4-fold change) were not induced in *Toxoplasma*-infected cells in response to IFN-γ (Figure 3A). Genes that were up-regulated in non-infected cells mainly clustered into biological processes related to immunity, and *Toxoplasma* infection decreased the number of genes within all clusters considerably (Figure 3B). Furthermore, those IFN-γ-inducible genes that were repressed by *T. gondii* were significantly enriched in processes involved in all aspects of the immune system, but also other processes, e.g. lipid catabolism, signal transduction and metabolic processes (Table S3A). On the other hand, 86% (n = 436) of the genes or transcripts that were repressed by IFN-γ in non-infected BMMΦ (IFN/Ctr ≤ 0.25), were not regulated in infected cells treated with IFN-γ (Figure 3A). Furthermore, 3% (n = 14) of genes that were repressed in non-infected BMMΦ in response to IFN-γ were even up-regulated in cells infected with the parasite (Figure 3A). Genes that were down-regulated after stimulation of non-infected cells with IFN-γ clustered into diverse biological processes, including both immune-related and immune-unrelated functions (Figure 3C). After concomitant infection with *T. gondii*, most of the IFN-γ-repressed processes appeared to be heavily disturbed. In addition, genes which were down-regulated by IFN-γ in non-infected BMMΦ, but counteracted after parasite infection were significantly enriched in several immune-related processes, but also a large number of immune-unrelated processes (Table S3B). This indicates that *Toxoplasma* not only targets IFN-γ-regulated immune mechanisms, but also other processes that are regulated by this cytokine. Nevertheless, the profound effect of infection on the macrophage transcriptome was highly specific for the response to IFN-γ. Only 1.3% (n = 518) of genes were up- or down-regulated after parasitic infection which were not regulated by IFN-γ (Figure 3A). Taken together, these data show that infection with *T. gondii* renders murine BMMΦ largely unresponsive to stimulation with IFN-γ, thereby preventing the activation of various processes, which are mostly but not exclusively related to host immunity.

### Defective assembly of the chromatin remodelling complex at GAS-containing sequences

We have shown previously that the IFN-γ-induced trafficking of STAT1 into the nucleus of *Toxoplasma*-infected murine macrophages or fibroblasts occurs as in non-infected controls [19] and this was confirmed herein (see below) as well as by others using human fibroblasts [22]. Since STAT1-induced transcriptional activation was nevertheless inhibited by parasite infection [19], we hypothesized a defect in chromatin remodelling and/or activation of the basal transcriptional machinery.

**Figure 4:**
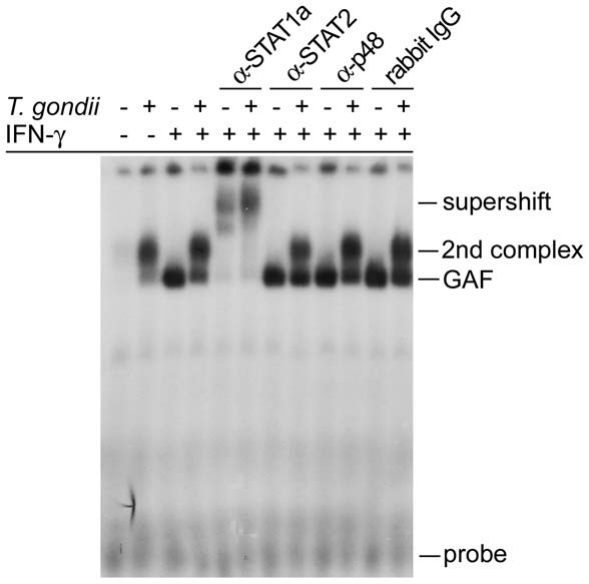
Binding activity of STAT1 homodimers to GAS-containing DNA sequences is modulated after *Toxoplasma* infection. Murine BMMΦ were infected or not with *T. gondii* for 24 hours, and were stimulated with IFN-γ during the final 30 minutes, or were left non-stimulated. Nuclear extracts were tested for their binding to radiolabelled GAS-containing oligonucleotides by electromobility shift assay. Supershift assays were performed using antibodies against STAT1α, STAT2 or p48, or with normal rabbit IgG as a control. GAF: gamma activated factor.

To test this hypothesis, we first assayed nuclear extracts from *Toxoplasma*-infected macrophages for their ability to bind GAS-containing oligonucleotide probes. Activation with IFN-γ for 30 minutes induced strong binding of nuclear components from non-infected BMMΦ to a GAS consensus sequence (Figure 4). Addition of an anti-STAT1α rabbit antibody, but not anti-STAT2, anti-p48 or normal rabbit IgG supershifted the complex, thus suggesting that the binding activity represented STAT1 homodimers, i.e. the gamma-activated factor (GAF). However, when cells had been previously infected with *T. gondii*, GAF formation was strongly decreased as also reported previously [16]. Remarkably, we identified a second GAS-binding complex with reduced electromobility that specifically appeared in nuclear extracts from *Toxoplasma*-infected BMMΦ, irrespective of whether cells were activated with IFN-γ or not (Figure 4). The parasite-induced complex was also supershifted by anti-STAT1, but none of the other antibodies tested. Together, these results suggested that a GAS-binding complex of different composition than GAF is induced in *Toxoplasma*-infected BMMΦ.

**Figure 5:**
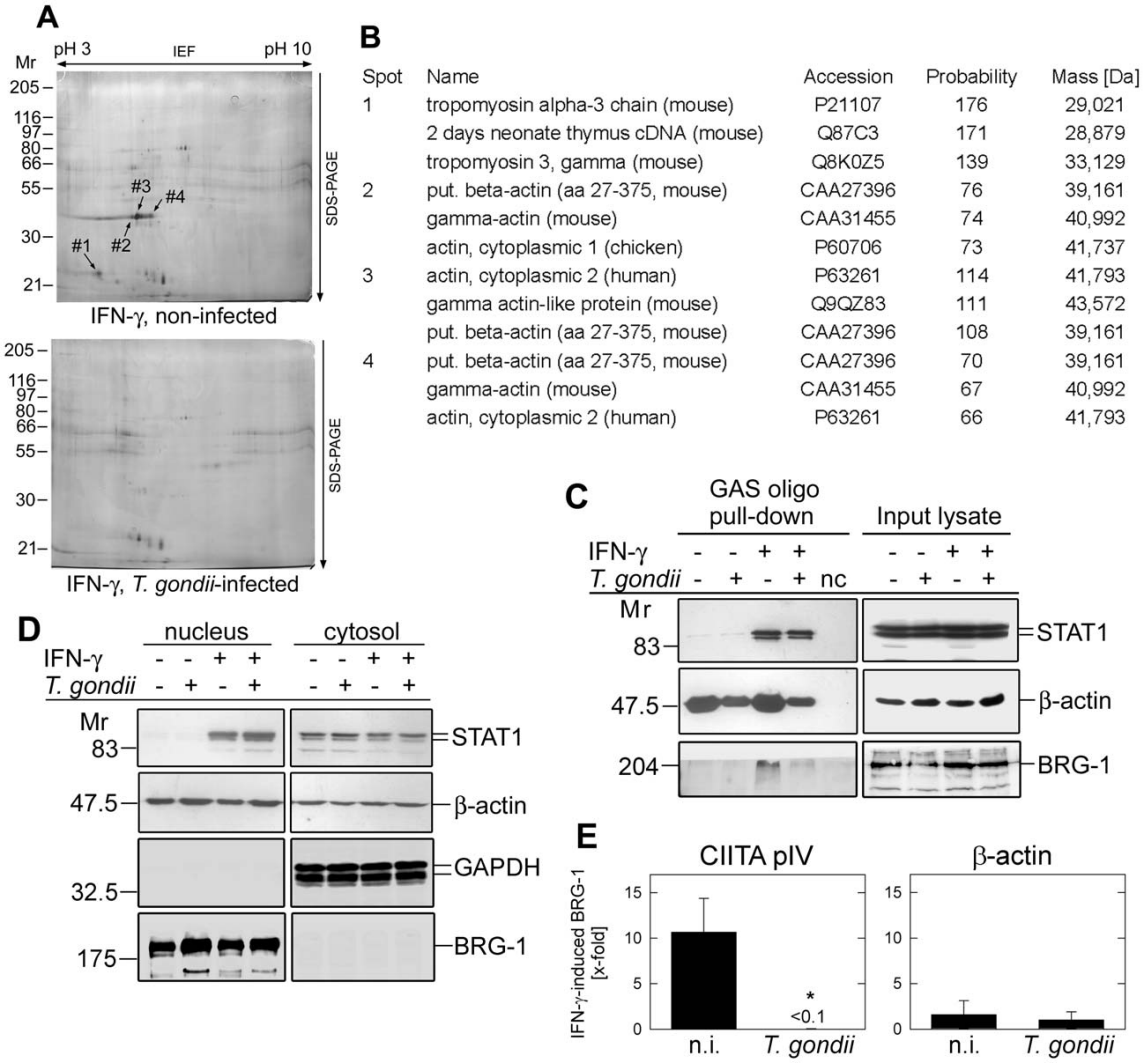
*Toxoplasma* infection leads to defective binding of components of chromatin-remodelling complexes to GAS-containing oligonucleotides *in vitro* and to the IFN-γ responsive CIITA promoter IV in infected cells. (A) RAW264.7 macrophages were infected with *T. gondii* for 24 hours, or were left non-infected and stimulated with IFN-γ during the final 30 min. Complete cellular lysates were used to isolate GAS-binding protein complexes by a pull-down assay. Precipitates were resolved by isoelectric focussing and SDS-PAGE, and proteins were visualized by silver staining. Protein spots that were specifically identified in the pull-downs from IFN-γ-treated, non-infected MΦ are indicated. (B) GAS-binding proteins that were pulled down from lysates of non-infected, but not *Toxoplasma*-infected MΦ after stimulation with IFN-γ were analysed by mass spectrometry. The three hits of highest probability for each of the four protein spots are shown; probability scores greater than 61 are considered significant. (C) RAW264.7 macrophages infected or not with *T. gondii* for 24 hours were stimulated with IFN-γ during the final 30 min, or were left non-stimulated. Complete cellular extracts or proteins that had been pulled-down from the extracts, by using a biotin-conjugated GAS-containing oligonucleotide and immobilized streptavidin, were subsequently analysed by immunoblotting using antibodies directed against STAT1, β-actin and BRG-1. Nc: negative control: pull-down assay without lysate. (D) Nuclear and cytosolic extracts were prepared from RAW264.7 macrophages infected with *T. gondii*, and stimulated with IFN-γ as described in (C). After separation of the extracts by SDS-PAGE, STAT1, β-actin, GAPDH and BRG-1 were detected by immunoblotting. (E) *Toxoplasma*-infected and non-infected (n.i.) RAW264.7 macrophages were stimulated with IFN-γ for 2 hours, or were left non-stimulated. After cross-linking DNA-protein complexes and shearing, chromatin was immunoprecipitated using an anti-BRG-1 antibody and protein A agarose. After DNA isolation from immunoprecipitates or from input lysates, fragments of the CIITA promoter IV (pIV) or β-actin were amplified by real-time PCR. The IFN-γ-induced binding of BRG-1 was calculated according to the ratio (E_ChIP_)^ΔCPChIP(unstimulated – IFN-γ-treated)^/(E_input_)^ΔCPinput(unstimulated – IFN-γ-treated)^. Results are means ± S.E.M. (n = 2), significant differences were identified by Student's *t*-test (*, *p*<0.05).

In an attempt to characterize differences in their protein composition, GAS-binding complexes from *Toxoplasma*-infected and non-infected macrophages were pulled-down, and were separated by two-dimensional gel electrophoresis. Since initial attempts failed to isolate sufficient material from nuclear extracts of infected and non-infected cells for 2D gel electrophoresis and subsequent mass spectrometry, we used complete cell extracts of RAW264.7 monocytes/macrophages for these analyses. Following silver staining of the 2D gels, several proteins were identified which had been specifically pulled-down from non-infected, but not *T. gondii*-infected MΦ after IFN-γ treatment (Figure 5A). In contrast, no proteins were detected, which were only present in GAS oligonucleotide precipitates from parasite-infected MΦ treated with IFN-γ. Protein spots which were specifically identified in IFN-γ-treated, non-infected MΦ (indicated in Figure 5A by arrows) were subsequently subjected to mass spectrometry. Mass finger print analyses identified tropomyosin 3 and putative beta-actin (syn. actin, cytoplasmic 1) or gamma-actin (syn. actin, cytoplasmic 2) as the hits with the highest probability (Figure 5B). Importantly, non-muscle actin is a constitutive component of chromatin remodelling complexes, and is required for RNA polymerase II-driven transcription [40]–[43]. Nuclear myosin 1 also plays important roles in transcription by nuclear RNA polymerases [43], and tropomyosin has been recognized in nuclei of human cells [44], although its nuclear function remains unknown. From the above results it was thus tempting to speculate that infection with *T. gondii* inhibited the assembly of chromatin remodelling complexes and/or the basal transcription apparatus at GAS-containing promoters following IFN-γ treatment. To further corroborate this hypothesis, GAS oligonucleotide pull-down assays were performed using extracts from *Toxoplasma*-infected and non-infected RAW264.7 cells, before and after treatment with IFN-γ, and were analysed by immunoblotting. The results showed that STAT1 associated with GAS sequences in an IFN-γ-dependent manner, and that the amount of precipitated STAT did not differ between infected and non-infected cells (Figure 5C). This confirmed those data obtained by EMSA showing that STAT1 protein complexes from *Toxoplasma*-infected cells in principal do bind to GAS sequences, although mostly at lower electromobility as compared to GAF (Figure 4), which was, however, not retained under denaturing condition (Figure 5C). Remarkably, β-actin recruitment to GAS oligonucleotides was severely impaired after parasite infection, and this was similarly observed whether cells were treated with IFN-γ or not. This indicated that actin binding to GAS oligonucleotides occurs independently of STAT1. BRG-1 is a core subunit of chromatin remodelling complexes. It has nuclear ATPase activity and is required for full activity of the complex [33]. In order to confirm a defective recruitment of chromatin remodelling components in lysates from *Toxoplasma*-infected MΦ, we also assayed GAS oligonucleotide precipitates for complex formation with BRG-1. The results showed its recruitment in non-infected macrophages in an IFN-γ-dependent manner, however, such binding was considerably impaired in lysates from infected cells (Figure 5C). As expected, immunoblotting of input lysates revealed no differences in the abundance of total STAT1, β-actin or BRG-1 between infected cells and non-infected controls, or between cells treated or not with IFN-γ (Figure 5C). Furthermore, the subcellular localization of β-actin also remained unchanged following infection or IFN-γ treatment, as determined by immunoblotting of nuclear and cytosolic extracts (Figure 5D) as well as by immunofluorescence staining and confocal microscopy (data not shown). In contrast, nuclear localization of STAT1 required previous activation of the cells with IFN-γ, and parasite infection did not interfere with this STAT1 redistribution as described previously [19]. Control staining of nuclear BRG-1 and cytosolic GAPDH confirmed the fractionation of macrophages into nuclear and cytosolic extracts (Figure 5D).

We next asked whether *Toxoplasma* infection also inhibits the assembly of chromatin remodelling complexes at IFN-γ-responsive promoters in infected cells. To this end, the recruitment of BRG-1 as a core subunit of chromatin remodelling complexes to the IFN-γ-regulated CIITA promoter IV (CIITA pIV) was assessed by chromatin immunoprecipitation (ChIP). Importantly, following stimulation with IFN-γ, BRG-1 increased 10-fold at the CIITA pIV of non-infected cells, but was hardly detectable at CIITA pIV of *T. gondii*-infected cells (Figure 5E; *p*<0.05, Student's *t*-test). In contrast, the abundance of BRG-1 did not significantly change at ß-actin chromatin in infected or non-infected MΦ in response to IFN-γ. Together, these results suggested that *T. gondii* infection of MΦ leads to an altered formation of STAT1-GAS complexes in response to IFN-γ, and to a defective assembly of chromatin remodelling complexes at STAT1-responsive elements *in vitro* and *in vivo*.

### Defects of histone acetylation at IFN-γ-responsive promoters following infection

**Figure 6:**
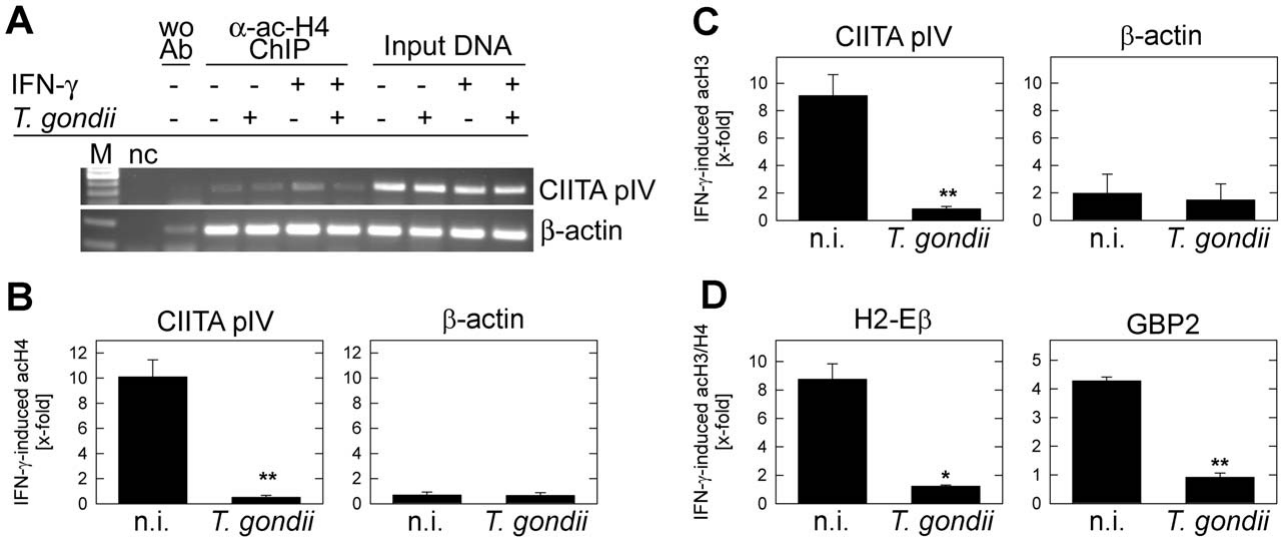
*Toxoplasma* infection impairs IFN-γ-induced acetylation of histones H3 and H4 at IFN-γ-responsive promoters in murine macrophages. (A) RAW264.7 macrophages were infected with *T. gondii* for 24 hours or were left non-infected, and were stimulated or not with IFN-γ during the final 16 hours. After cross-linking DNA-protein complexes, cell lysates were subjected to ChIP analysis using an anti-acetyl-H4 antibody and protein A agarose. A no-antibody control was run in parallel (wo Ab). After isolation of DNA from chromatin immunoprecipitates or from input cell lysates, fragments of the CIITA pIV or β-actin were amplified by real-time PCR. Amplicons were verified by agarose gel electrophoresis. Nc: PCR negative control: without template. (B+C) Acetylation of histones H4 (B) and H3 (C) at the CIITA pIV and β-actin after stimulation of MΦ with IFN-γ was analysed by ChIP as described above and was quantified according to the ratio (E_ChIP_)^ΔCPChIP(unstimulated – IFN-γ-treated)^/(E_input_)^ΔCPinput(unstimulated – IFN-γ-treated)^. Results are means ± S.E.M. from three independent experiments, significant differences between the acetylation in non-infected (n.i.) and infected (*T. gondii*) MΦ were identified by Student's *t*-test (**, *p*<0.01). (D) The histone H3/H4 acetylation at the H2-Eβ and the GBP2 promoters of non-infected and *Toxoplasma*-infected MΦ was analysed by ChIP as described above (n = 2).

Actively transcribed genomic regions are characterized by post-transcriptional modifications, including the acetylation of N-terminal lysine residues of the core histones H4 and H3. Acetylated histones facilitate euchromatin formation and assembly of the transcriptional machinery on promoter DNA [32], [45]. Hyperacetylation of histones H3 and H4 within the respective promoter chromatin is also required for the IFN-γ-induced up-regulation of MHC class II or interferon-induced guanylate-binding protein 2 (GBP2) [34], [36]. Therefore, we next examined the possibility of defective histone acetylation in *Toxoplasma*-infected MΦ following IFN-γ treatment using ChIP analysis. The results showed an increase in the acetylation of histone H4 at the CIITA pIV, following stimulation of non-infected MΦ with IFN-γ, however, such increase was not observed in *Toxoplasma*-infected MΦ (Figure 6A). Neither control amplifications of CIITA pIV using input DNA as a template, nor those of β-actin from anti-ac-H4 ChIP precipitates revealed differences between infected and/or IFN-γ treated cells. Importantly, quantification by real-time PCR indicated that infection with *T. gondii* abolished the IFN-γ-induced histone H4 acetylation of CIITA pIV chromatin by more than 90% (Figure 6B; *p* = 4×10^−7^; Student's *t*-test). The extent of inhibition of histone H4 acteylation concorded with the decrease of IFN-γ-regulated CIITA mRNA after parasitic infection, as observed herein by microarray analyses (IFN-γ-induced CIITA mRNA: 32.04 in non-infected MΦ vs. 3.17 in *Toxoplasma*-infected MΦ, see Table S1), or as previously shown by northern blotting [16]. It is also in agreement with the inhibition of CIITA pIV activity, as revealed by luciferase reporter assay [19]. In contrast to CIITA pIV chromatin, no significant difference in the amount of acetylated histone H4 was recognized between β-actin chromatin from infected as compared to that from non-infected MΦ (Figure 6B). Likewise, the IFN-γ-induced acetylation of histone H3 at CIITA pIV, but not at β-actin was significantly reduced following infection with *T. gondii* (Figure 6C; *p* = 0.006). Finally, the acetylation of histones H3 and H4 in infected MΦ was also impaired at the promoters of the IFN-γ-regulated genes *H2-Eβ* and *gbp2* (Figure 6D). It has to be stressed that the level of parasite-imposed inhibition of histone acetylation differed between CIITA pIV and H2-Eβ on the one hand and GBP2 on the other suggesting promoter-specific effects. Together, the data nevertheless clearly indicate severely impaired chromatin remodelling at least at distinct IFN-γ-responsive promoters in *T. gondii*-infected macrophages.

### HDAC inhibitors restore defective IFN-γ responses in *Toxoplasma*-infected MΦ

**Figure 7:**
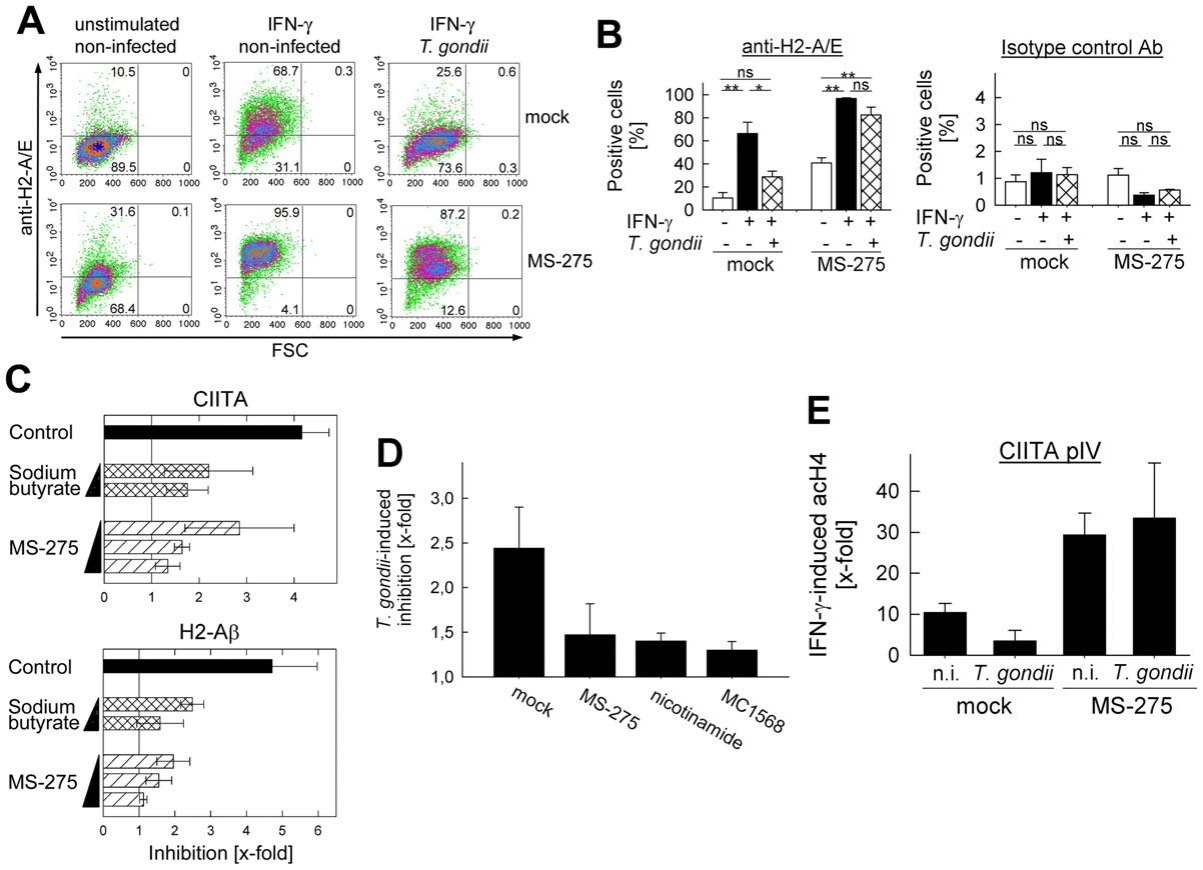
HDAC inhibitors restore responsiveness of *Toxoplasma*-infected macrophages to IFN-γ. (A) RAW264.7 macrophages were infected with *T. gondii* (parasite-host cell ratio 3∶1), or were left non-infected and stimulated with IFN-γ in the absence (upper panel) or presence of 2 µM of the HDAC inhibitor MS-275 (lower panel). Forty hours after infection, cells were immunolabelled with anti-H2-A/E and analysed by flow cytometry. Results are from a representative experiment out of three, data are the percentages of cells within the individual quadrants. (B) Murine macrophages were infected with *T. gondii* and/or treated with IFN-γ and MS-275 as described in (A). Cells were immunolabelled with an antibody directed against H2-A/E or an isotype-matched control antibody, and were analysed by flow cytometry (please consider the different scaling of the x-axes in both plots). Data represent the mean percentages ± S.E.M. of positive cells from three independent experiments. *: *p*<0.05, **: *p*<0.01, ns: not significant (Student's *t*-test). (C) IFN-γ-induced transcript levels of CIITA and H2-Aβ in infected and non-infected RAW264.7 macrophages after treatment with HDAC inhibitors MS-275 or sodium butyrate. Cells were infected or not with *T. gondii* for 24 hours, and treated with IFN-γ in the absence (control) or in the presence of 0.5 or 2 µM sodium butyrate or 0.5, 1 or 2 µM MS-275 as indicated. After isolation of RNA and reverse transcription of mRNA, CIITA, H2-Aβ and β-actin were amplified, and semi-quantitatively analysed. IFN-γ-induced CIITA and H2-Aβ transcript levels in *T. gondii*-infected and non-infected samples were normalized to β-actin, and the fold inhibition imposed by parasitic infection was calculated. Data represent means ± S.E.M. from three independent experiments. (D) Impact of HDAC inhibitors targeting different HDACs on *Toxoplasma*-mediated inhibition of H2-A/E expression. Murine macrophages were infected with *T. gondii* and/or treated with IFN-γ and MS-275, MC1568 (both at 2 µM) or nicotinamide (5 mM) as described in (A). After immunolabelling with an antibody directed against H2-A/E, cells were analysed by FACS. The parasite-imposed inhibition of IFN-γ-induced H2-A/E expression was calculated; data represent means ± S.E.M. from three independent experiments. (E) Acetylation of histone H4 at the CIITA pIV in the presence or absence of MS-275. *Toxoplasma*-infected and non-infected MΦ (n.i.) were treated or not with 2 µM MS-275, and stimulated with IFN-γ or left non-stimulated. After cross-linking DNA-protein complexes, cell lysates were analysed by ChIP using an anti-acetyl-H4 antibody. ChIP and input DNA were amplified by real-time PCR using primers specific for the CIITA pIV. The IFN-γ-induced H4 acetylation in the presence or absence of MS-275 was quantified according to the ratio (E_ChIP_)^ΔCPChIP(unstimulated – IFN-γ-treated)^/(E_input_)^ΔCPinput(unstimulated – IFN-γ-treated)^. Results are means ± S.E.M. from two independent experiments.

The activities of histone acetyl transferases (HATs) and histone deacetylases (HDACs) govern the acetylation of histones, and are crucial regulators of transcriptional activation and repression [32]. Aberrant histone modifications may result from an imbalance of these modifying enzymes. HDAC inhibitors are being extensively studied for their ability to counteract hypoacetylation of histones in malignant cells, and they have been approved or are being considered as epigenetic cancer therapies [46]. Since the defective IFN-γ response of *T. gondii*-infected MΦ coincided with an inability to hyperacetylate histones H3 and H4 at IFN-γ-responsive promoters, we reasoned that HDAC inhibitors could potentially restore IFN-γ responses in infected cells. In order to test this hypothesis, we determined H2-A/E expression in infected and non-infected MΦ treated or not with the HDAC inhibitor MS-275 by fluorescence activated cell sorting (FACS). After treatment with IFN-γ, non-infected cells significantly up-regulated the expression of H2-A/E molecules (*p*<0.01; Student's *t*-test) on the cell surface, however, concomitant infection with *T. gondii* diminished such up-regulation to a large extent (Figure 7A, upper panel). H2-A/E expression in parasite-infected cells after stimulation with IFN-γ did indeed not differ significantly from that in non-stimulated controls (Figure 7B; *p* = 0.51). Infection with GFP-expressing mutants of *T. gondii* showed that IFN-γ-induced H2-A/E expression was decreased in both parasite-positive and parasite-negative cells of an infected MΦ population (Figure S2). This result came unexpected since previous reports have suggested that IFN-γ-induced gene expression is predominantly inhibited in parasite-positive cells of a *Toxoplasma*-infected host cell population [15], [22]. It will be interesting to address this issue in the future and to re-evaluate a possible involvement of a soluble factor either derived from the parasite or the host cell. Both type II PTG and type I RH parasites clearly diminished H2-A/E expression in response to IFN-γ although strain-specific differences in the levels of suppression became apparent (Figure S2). Treatment of MΦ with 2 µM MS-275 prior to IFN-γ stimulation potentiated H2-A/E up-regulation in non-infected cells (Figure 7A, lower panel) as already suggested by the findings of others [36]. Most importantly, MS-275 also enabled *Toxoplasma*-infected MΦ to respond to IFN-γ, as revealed by the significant up-regulation of H2-A/E molecules following stimulation (Figure 7B; *p*<0.01). Infection with GFP-expressing parasites revealed that MS-275 restored H2-A/E expression in parasite-positive and parasite-negative cells of an infected macrophage population (Figure S2). Furthermore, cells harbouring type I RH or type II PTG parasites were rescued following treatment with MS-275. Consequently, IFN-γ-regulated H2-A/E expression did not differ significantly between *Toxoplasma*-infected and non-infected MΦ in the presence of MS-275 (Figure 7B; *p* = 0.1). Control staining with an isotype-matched control antibody did not reveal any significant fluorescence, and did not differ between the various samples (Figure 7B). In addition, prior control experiments showed that MS-275 at the concentrations used in these experiments had no adverse effect on infectivity or intracellular replication of *T. gondii* (Figure S3). Replication was also observed in MS-275-treated macrophages in the presence of IFN-γ suggesting that these parasites employ additional mechanisms to resist IFN-γ-mediated cell autonomous immunity different from those employed to interfere with IFN-γ-induced gene expression such as H2-A/E. These might be similar but not identical to those recently described for type I parasites [47]–[48]. Together, these results clearly showed that MS-275 was able to restore defective H2-A/E expression in *Toxoplasma*-infected MΦ.

RT-PCR analyses further corroborated the FACS data. The transcript levels of H2-Aβ strongly increased after activation of non-infected MΦ with IFN-γ, whereas prior infection with *Toxoplasma* inhibited such increase 4.7-fold (Figure 7C). Importantly, the HDAC inhibitors MS-275 or sodium butyrate at 0.5 to 2 µM both diminished the *Toxoplasma*-imposed inhibition of H2-Aβ transcripts in a dose-dependent manner, and rescued infected MΦ almost completely from their inability to up-regulate H2-Aβ at the highest concentration used. Similarly, the defective up-regulation of CIITA as observed after infection of IFN-γ-treated MΦ with *T. gondii* was also dose-dependently diminished by sodium butyrate and MS-275 (Figure 7C). Two µM sodium butyrate did not diminish the intracellular replication of *T. gondii* as already shown for MS-275 (Figure S3). Together, this suggests that IFN-γ-responsive promoters in general, including those directly depending on STAT1 binding, can be rendered responsive, when treating *Toxoplasma*-infected MΦ with HDAC inhibitors.

Mammals express a variety of different HDACs, which are classified according to biochemical features and expression patterns. They belong to one of four different classes, i.e. the classical HDAC classes I, II and IV and the NAD^+^-dependent sirtuins (also designated class III HDACs) [49]. HDACs associate with different co-regulators, and have different substrate specificities. It was thus of further interest, whether we could identify specific HDACs that are of particular relevance for *Toxoplasma*-imposed blockade of IFN-γ-induced gene expression in MΦ. To address this question we employed HDAC inhibitors with selective substrate specificities, and measured their impact on IFN-γ-induced H2-A/E expression. The results showed that MS-275 (inhibits class I HDACs except HDAC 8 [50]) and MC 1568 (inhibits class II HDACs [50]) increased the IFN-γ-regulated H2-A/E expression of *Toxoplasma*-infected macrophages considerably (Figure S4). Nicotinamide (inhibits sirtuins [51]) increased H2-A/E expression of infected cells only slightly, but it considerably decreased that of non-infected cells, and therefore the net increase in infected and nicotinamide-treated cells was considerable compared to infected and mock-treated cells. Consequently, after treatment with the inhibitors tested, H2-A/E expression of infected and non-infected MΦ did not differ significantly as observed in mock-treated cells (Figure S4B). Finally, MS-275, nicotinamide and MC1568 all diminished *Toxoplasma*-mediated decrease in IFN-γ-regulated H2-A/E expression to a similar extent (Figure 7D). As already shown for MS-275, nicotinamide did not affect parasite replication whereas MC1568 diminished, but did not abolish parasite propagation (Figure S3).

We finally asked the question whether HDAC inhibition acts on *Toxoplasma*-infected cells by increasing chromatin remodelling at IFN-γ-regulated promoters. To this end, histone H4 acetylation at the CIITA pIV was analysed in MΦ treated or not with MS-275 by ChIP. The results showed that MS-275 increased IFN-γ-triggered acetylation of histone H4 in both non-infected and *T. gondii*-infected cells, but the increase was clearly higher in infected cells (9.7-fold) than in non-infected cells (2.8-fold; Figure 7E). Thus, whereas there was a significant reduction in IFN-γ-induced H4 acetylation in *T. gondii*-infected cells as compared to non-infected cells, this change was abolished in cells concomitantly incubated with MS-275. Together, these results indicate that by decreasing the activity of HDACs, and increasing histone acetylation at IFN-γ-responsive promoters, *Toxoplasma*-infected murine MΦ can be rescued from the parasite-imposed unresponsiveness to IFN-γ.

## Discussion

Infections of mice or humans with *T. gondii* elicit a robust inflammatory response with secretion of substantial levels of IFN-γ by CD4^+^, CD8^+^ T lymphocytes and NK cells [5]–[6], [11]–[14]. IFN-γ production during toxoplasmosis is instrumental in orchestrating and exerting cell-mediated immunity towards the parasite [3], [7], [12], [30], and it is therefore at first sight surprising, that even high IFN-γ responses do not suffice to eradicate *T. gondii* [13]–[14]. Using a genome-wide transcriptome analysis, we here show that *Toxoplasma* renders murine MΦ largely unresponsive to stimulation with IFN-γ. We consider this a crucial mechanism of the parasite to establish a productive infection in the immune-competent host.

Primary murine MΦ differentially regulated the expression of 763 unique genes in response to IFN-γ, thus confirming the highly pleiotropic activity of this cytokine [9]–[10]. Concomitant infection with *T. gondii*, however, abrogated the expression pattern of the majority of IFN-γ-induced or IFN-γ-repressed genes. It is important to note, that interference of *T. gondii* with MΦ gene expression is highly specific towards IFN-γ-regulated genes. Expression of only 1.3% of genes not regulated by IFN-γ was altered in parasite-infected cells as compared to non-infected control cells. This rules out a general transcriptional defect of *Toxoplasma*-infected MΦ, and thus confirms previous findings on the repression of individual IFN-γ-regulated genes by *T. gondii* infection [15], [18], [20] on a genome-wide level. It also strongly suggests that *T. gondii* specifically targets the JAK/STAT signalling pathway, or the transcriptional activation in response to IFN-γ. In addition to abrogating the expression of a large subset of IFN-γ-regulated genes, *T. gondii* considerably counteracted the expression levels of the vast majority of the remaining IFN-γ-regulated genes.

Consistent with the severe defect to respond to IFN-γ, a wide variety of biological processes was abolished in *Toxoplasma*-infected MΦ as revealed by network analyses (see Table S3). These included predominantly processes involved in regulatory and effector functions of immunity, as it could be expected from the main functions of IFN-γ [9]. Remarkably, we also recognized several biological processes being significantly affected by parasite infection, which are at first sight not directly linked to the immune system, e.g. skeletal system development, signal transduction, membrane organization and lipid transport (see Table S3, Figure 3). The impact of counteracting IFN-γ-regulated gene expression in MΦ on the host-pathogen interplay might thus be broader than previously unravelled after analysing single immunity-related genes [15]–[18], [20]. However, the biological significance of parasite interference with such processes still remains to be determined. We also need to consider the critical anti-parasitic activity of IFN-γ during toxoplasmosis [3], [7], [12], [30] as the main driving force for *T. gondii* to evolve mechanisms for its efficient inhibition.

It should be stressed that *T. gondii* parasites do not completely abolish IFN-γ-responses of murine MΦ. Several of those genes induced or repressed by IFN-γ in non-infected cells were still differentially regulated by IFN-γ in infected cells, although mostly at a significantly reduced level. In contrast to human fibroblasts [22], murine MΦ thus retain some residual responsiveness to IFN-γ after *Toxoplasma* infection. As discussed above this may relate to the critical function of MΦ in immunity and their stronger responsiveness to IFN-γ compared to fibroblasts. We also identified several genes, e.g. *CXCL11*, *CFB*, *CD69*, *FLT1*, *CD14* or *MMP9*, the regulation of which was even amplified by *T. gondii* infection (see Tables S1 and S2). Such synergism was in some cases (e.g. *CD69*, *IFIT2*, *MX1*) attributed to additive effects on gene regulation induced by *Toxoplasma* infection independently of IFN-γ. Mostly though, it required the concomitant presence of both parasite and IFN-γ. This indicates a complex differential impact of *T. gondii* infection on MΦ gene expression that clearly differs depending on the cytokine milieu. The molecular basis of the differential regulation is not entirely clear, but may be due to the additive or cooperative effect of IFN-γ, and/or other factors due to *T. gondii* infection. Importantly, residual IFN-γ-responsiveness of *Toxoplasma*-infected MΦ, and parasite regulation of IFN-γ-responsive genes may be critical to limit parasite replication at least to some extent, and to favour host survival during acute toxoplasmosis. This assumption is consistent with findings that mice treated with IFN-γ-depleting monoclonal antibodies, or with a targeted disruption of the *IFN-γ* or *IFN-γR* genes rapidly succumb due to acute toxoplasmosis or reactivation of chronic infection [3], [7], [12]. Similarly, the inability of immunocompromized humans to control a chronic *Toxoplasma* infection may be attributed to the loss of residual IFN-γ-regulated gene expression, when the T cell-mediated production of Th1-type cytokines including IFN-γ declines. Hence, we here propose that the large-scale evasion of IFN-γ responses by MΦ infected with *T. gondii* on the one hand and the residual IFN-γ regulated gene expression on the other hand are crucial for establishment of a balanced parasite-host interaction during toxoplasmosis.

Interference of *T. gondii* with IFN-γ-mediated gene expression on a global scale suggests a common underlying mechanism. This was not self-evident since IFN-γ not only activates the classical JAK/STAT signalling pathway, but also the MEK-ERK pathway, PI3K, G-protein linked C3G and Rap-1, Fyn kinase, SHP-1 and -2 protein tyrosine phosphatases, as well as CRK adaptor proteins [31], [52]. Consequently, a remarkably large number of genes are differentially regulated in STAT1^-/-^ BMMΦ following IFN-γ treatment [53]. Our study nevertheless revealed a highly significant correlation between the levels of gene regulation that were elicited by IFN-γ and the parasite's impact on the IFN-γ response. This further argues for a common mechanism of *T. gondii* to counteract IFN-γ-regulated gene expression in MΦ. It should be stressed that we herein mainly used a mouse-avirulent type II strain (NTE), i.e. a strain of the clonal lineage, which is most commonly found in Europe and North America. Whereas type I and type III strains of *Toxoplasma* have been clearly shown to also inhibit IFN-γ-regulated gene expression of their host cells [20], [22], we cannot currently rule out additional mechanisms of these strains to inhibit the IFN-γ response of their host cells. Using mouse-virulent type I parasites, Zimmermann et al. suggested the induction of SOCS-1, and consequently a decrease in STAT1 tyrosine phosphorylation within the host cell cytosol, leading to defective IFN-γ-responses of MΦ following infection [20]. Whereas this was not confirmed in human fibroblasts, Kim and colleagues instead implicated a dephosphorylation of nuclear STAT1 in particular following infection with type I strain parasites [22]. These results consistently indicate that mouse-virulent type I *Toxoplasma* strains may have evolved additional means to inhibit IFN-γ responses of the host cell.

**Figure 8:**
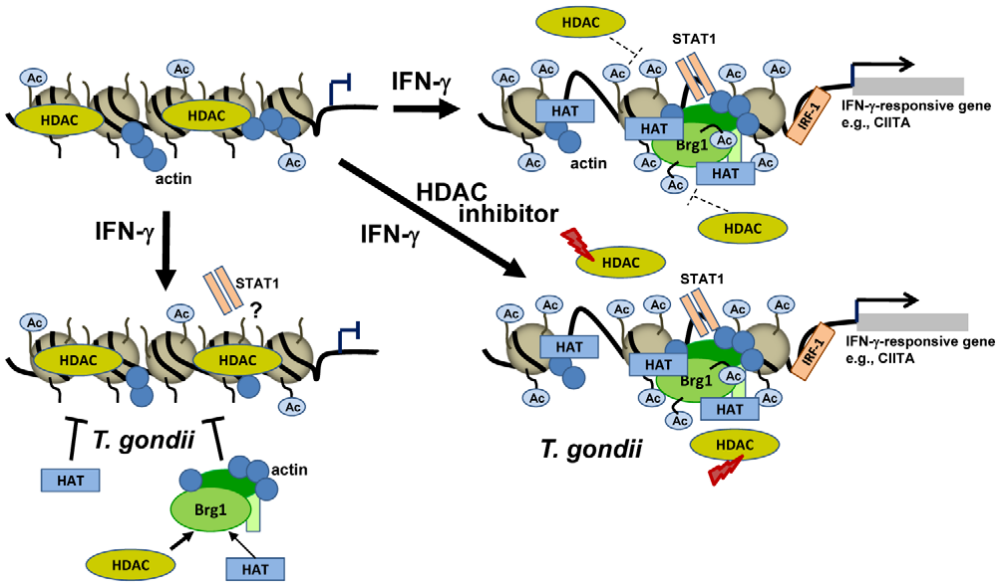
Model of *Toxoplasma*-imposed inhibition of IFN-γ-mediated gene expression, and the rescue of murine MΦ from such unresponsiveness using HDAC inhibitors. In the absence of IFN-γ, hypoacetylated histones due to predominant HDAC activity lead to compact chromatin and repression of promoter activation (upper left). After stimulation with IFN-γ, recruitment of BAF including the ATPase BRG-1 leads to changes in chromatin structure that allows subsequent binding of STAT1 to its consensus sequence, recruitment of HATs, and hyperacetylation of histones and non-histone proteins. Recruitment of BAF as well as full activity of BRG-1 and possibly other components of active STAT1-regulated promoters require the presence of non-muscle actin. Finally, binding of IRF-1 allows binding of the transcriptional machinery and Pol II-driven gene transcription (upper right). In *Toxoplasma*-infected MΦ, reduced levels of actin at STAT1-responsive promoters coincide with reduced recruitment of chromatin remodelling complexes, including BRG-1 and HATs and thereby favour hypoacetylated chromatin. STAT1 does either aberrantly bind to such promoters, or does not bind in infected cells (lower left). Treatment of *Toxoplasma*-infected MΦ with HDAC inhibitors leads to a shift towards increased acetylation of histones and/or non-histone proteins, and thereby favours the switch to permissive chromatin following IFN-γ activation. This facilitates binding of transcription factors and chromatin remodellers in the presence of *T. gondii*, and allows binding of the transcriptional machinery and IFN-γ-induced gene expression (lower right).

Herein, we present several independent lines of evidence that *Toxoplasma* renders MΦ unresponsive to IFN-γ via an impairment of the chromatin remodelling at IFN-γ-responsive promoters: (i) recruitment of components of chromatin remodelling complexes to GAS-containing oligonucleotides and to IFN-γ-responsive promoters was disturbed following infection, (ii) GAF formation, i.e. binding of STAT1 homodimers to a GAS consensus sequence was inhibited after infection, (iii) the IFN-γ-induced acetylation of core histones at different IFN-γ-responsive promoters was strongly decreased in infected cells, and (iv) treatment with HDAC inhibitors rescued MΦ from the *Toxoplasma*-mediated inhibition of IFN-γ-induced gene expression (Figure 8). These results concur with previous findings that despite normal translocation of STAT1 into the nuclei of infected cells following IFN-γ treatment (see [19], [22] and Figure 5D, this study), transcriptional activation of a *luc* reporter gene under control of IFN-γ-responsive promoters was nevertheless impaired [19]. The signalling event that we now recognize to be altered in parasite-infected cells is an impaired binding of actin and BRG-1 to GAS-containing oligonucleotides. Importantly, recruitment of BRG-1, i.e. a core subunit of chromatin remodelling complexes like BAF to the IFN-γ-regulated CIITA pIV was also inhibited in infected cells. Non-muscle actin is another component of chromatin remodelling complexes, and facilitates or even mediates their binding to chromatin [40]. It also interacts with BRG-1, and is required for its full ATPase activity [40]. During activation of the prototypic IFN-γ-regulated promoters of CIITA and GBP2, recruitment of BAF including BRG-1 is required for subsequent binding of STAT1 and histone acetylation [34], [37]. EMSA indeed confirmed a diminished formation of GAF, i.e. binding of STAT1 dimers to GAS in *Toxoplasma*-infected MΦ as compared to non-infected controls. Furthermore, acetylation of histones H3 and H4 in response to IFN-γ was also diminished in infected cells.

Intriguingly, the impaired binding of actin to GAS sequences occurred independent of whether MΦ have been activated with IFN-γ and whether STAT1 or BRG-1 have bound to GAS. Actin may thus also be constitutively present at IFN-γ-responsive promoters with reduced levels being observed in *Toxoplasma*-infected cells. This result is remarkable, since it strongly supports the hypothesis of an interference of the parasite with IFN-γ-regulated gene expression, which occurs independently of the activity of STAT1. Kim et al. recently reported a partial decrease of Tyr^701^- phosphorylated STAT1 in the nuclei of human fibroblasts, but they also stressed that this observation cannot fully account for the inhibition of IFN-γ-regulated gene expression [22]. The impaired chromatin remodelling in *Toxoplasma*-infected MΦ following stimulation with IFN-γ as reported herein may represent this hitherto unknown mechanism that accounts for major parts of the inhibition of IFN-γ-regulated gene expression exerted by the parasite. The fact that defective IFN-γ-mediated gene expression of CIITA and MHC class II molecules in *Toxoplasma*-infected MΦ can be largely abolished by treatment with HDAC inhibitors also argues for impaired chromatin remodelling rather than STAT1 activity as the major mechanism to inhibit IFN-γ responses in infected MΦ (see below). A parasite-mediated defect in the recruitment of chromatin remodelling complexes following activation of MΦ is appealing, since it might also be responsible for impaired Ser^10^ phosphorylation and Lys^9/14^ acetylation of histone H3 at the TNFα and IL-10 promoters following LPS or LPS/FcγR stimulation, respectively [54]–[55].

Another major finding of our study is that defective IFN-γ-responses in *Toxoplasma*-infected MΦ can be restored using HDAC inhibitors. This not only argues for chromatin modifications being the primary target of *T. gondii* to counteract IFN-γ responses in MΦ (see above), but, more importantly, also opens up novel possibilities for anti-parasitic treatment. A main function of HDACs is to remove acetyl groups from histones, thereby counteracting the activity of HATs and repressing gene expression [49]. More recently, HDACs have been assigned additional functions including the repression of CIITA activity [36], or the co-activation of transcription of distinct genes including IFN-γ-dependent *IP-10* [56]. Such requirement of HDAC activity may be due to acetylation of non-histone proteins [57]. Importantly, inhibitors of HDAC have been proven or are considered promising chemotherapeutic agents for the treatment of several human diseases including different malignancies and neurodegenerative diseases, and have already been approved for use in humans or are in clinical trials [46]. Here we show that MS-275 or sodium butyrate, i.e. widely used small molecule HDAC inhibitors [50], [58] rescue *Toxoplasma*-infected murine MΦ from the inhibition of IFN-γ-regulated gene expression. Importantly, treatment of *Toxoplasma*-infected macrophages with MS-275 was accompanied by a vigorous increase in acetylation of core histone H4 at the IFN-γ-responsive CIITA pIV. This therefore suggests that it indeed acts by reversal of defective chromatin remodelling in infected cells. HDAC inhibition thus suffices to induce a switch from repressive towards permissive chromatin in *Toxoplasma*-infected MΦ, and allows IFN-γ-mediated gene expression (Figure 8). We do not yet know the exact acetylation target(s) which is crucial in rescuing the host cells. Recent analyses of the *in vivo* acetylome have identified 3600 distinct lysine residues on 1750 proteins being acetylated in mammalian cells [59]. Beside histones these include multiple proteins involved in chromatin remodelling, cytoskeleton reorganisation, acetylation and methylation [59]. Therefore, we can currently not exclude the possibility, that either in addition to or instead of histones, subunits of the chromatin remodelling complex BAF show an acetylation defect in *Toxoplasma*-infected cells, which is restored in the presence of HDAC inhibitors. Screening of several inhibitors that target the three major classes of HDACs, i.e. class I and class II HDACs as well as sirtuins [50]–[51], revealed that a broad range of HDACs mediate the parasite-imposed inhibition of IFN-γ responsiveness. This finding indeed argues for a scenario where inhibition of HDACs results in a more general hyperacetylation of both histones and chromatin remodellers that collectively allow transcriptional activation of IFN-γ-responsive genes despite of the presence of *T. gondii*. It should also be stressed that distinct HDAC inhibitors act on the parasite itself, and can exert potent anti-*Toxoplasma* activity [60]–[61]. Control experiments excluded that the HDAC inhibitors used in this study severely impair or even kill the parasite (Figure S3). However, we cannot currently completely rule out that HDAC inhibitors restore IFN-γ responsiveness of infected macrophages by more subtle alterations to *T. gondii*'s physiology, e.g. by altering the expression of distinct parasite effector molecules. The fact that HDAC inhibitors of different chemistry and specificities consistently restored IFN-γ responses in infected macrophages, nevertheless clearly argues for an effect on acetylation of host cell proteins rather than parasite proteins.

Since parasite-imposed inhibition of IFN-γ-induced expression of H2-A/E molecules and CIITA were consistently abolished after treatment with HDAC inhibitors, we currently assume that inhibiting HDAC activity abrogates the global unresponsiveness of *Toxoplasma*-infected MΦ to IFN-γ. Due to the limited number of genes investigated, we cannot, however, completely rule out that expression of a subset of IFN-γ-regulated genes remains completely or partially inhibited after *T. gondii* infection, even in the presence of HDAC inhibitors. Our finding nevertheless makes the disturbed chromatin remodelling at IFN-γ-regulated promoters following *Toxoplasma* infection a promising and feasible target for a supportive therapy of toxoplasmosis. Due to the critical importance of IFN-γ in the anti-parasitic immunity during *Toxoplasma* infections [3], [7], [12], [30], shifting the balance towards more efficient IFN-γ responses of MΦ and probably other host cells may indeed facilitate parasite control, or may even suffice to eradicate the infection. This would, to our knowledge, represent the first time that a pathogens' immune evasion strategy becomes a target for an improved treatment of an infectious disease.

## Materials and Methods

### Macrophages, parasites and infection

Primary bone marrow-derived macrophages (BMMΦ) were isolated from female BALB/c mice as described before [15]. Briefly, bone marrow cells were cultured in RPMI 1640 supplemented with 10% heat-inactivated FCS, 100 U/ml penicillin, 100 µg/ml streptomycin and 20% L929-conditioned medium as a source of M-CSF. After differentiation for 6 days, adherent BMMΦ were incubated in RPMI 1640 medium (as above) devoid of L929-derived supernatant. The murine leukaemia monocyte/macrophage cell line RAW264.7 (TIB 71; ATCC, Rockville, MD) was cultured in RPMI 1640 containing 4.5 g/L glucose, 10% FCS, 1 mM sodium pyruvate, 10 mM HEPES and antibiotics. Tachyzoites of the mouse-avirulent type II strain NTE [62] were propagated in L929 fibroblasts as described before [15]. Where indicated, GFP-expressing mutants of mouse-virulent type I parasites (RH-LDM) or of mouse-avirulent type II parasites (PTG/ME49) were used (kindly provided by Antonio Barragan, Stockholm, Sweden). Prior to infection of macrophages, parasites were isolated by differential centrifugation and thoroughly washed [19]. Unless stated otherwise, cells were infected at parasite-host cell ratios of 6∶1 and 3∶1 for 24 or 40 hours, respectively. Infected macrophages or non-infected controls were stimulated with 100 or 300 U/ml recombinant mouse IFN-γ (R&D Systems, Wiesbaden, Germany) starting at 2 – 3 or 23.5 hours p.i. as indicated. In some experiments, the following HDAC inhibitors were added at 2 hours prior to the stimulation with IFN-γ: 0.5 or 2 µM sodium butyrate, 2 µM MC1568, 5 mM nicotinamide (all from Sigma, Steinheim, Germany), or 0.5 to 2 µM N-(2-aminophenyl)-4-[N-(pyridine-3-ylmethoxycarbonyl)aminomethyl]benzamide (MS-275; Merck, Darmstadt, Germany).

### Microarray analysis

Twenty-four hours after infection, total RNA from *Toxoplasma*-infected or non-infected primary BMMΦ that had been cultured in the presence or absence of 100 U/ml IFN-γ was isolated using the RNeasy kit (Qiagen, Hilden, Germany). RNA quality was assured using the Agilent 2100 Bioanalyser (Agilent Technologies, Santa Clara, CA) before RNAs from 4 biological replicates each were pooled, and then treated with DNase I. After phenol/chloroform/isoamylalcohol extraction, isopropanol/sodium acetate precipitation and RNA quality control as above, mRNA was isolated using the Oligotex mini kit as recommended (Qiagen). CyDye-labelled cDNA probes were prepared using the CyScribe post-labelling kit, according to the manufacturer instructions (Amersham Biosciences, Freiburg, Germany). Briefly, cDNA was synthesized in the presence of amino allyl-dUTP using CyScript reverse transcriptase and both random nonamer and oligo(dT) primers. After purification, amino allyl-modified cDNA was chemically labelled with Cy3 or Cy5 NHS-esters. Labelled cDNA was purified using the GFX purification kit (Amersham Biosciences). Finally, quantity and dye incorporation rates were assessed using a NanoDrop ND-100. Per microarray, 0.3 µg Cy3 and Cy5-labelled cDNAs, respectively, were simultaneously hybridized for 17 hours at 63°C to Agilent Technologies 44K mouse whole genome 60-mer oligonucleotide microarrays representing ∼41,000 mouse genes and transcripts. Slides were washed as recommended (Agilent Technologies SSPE protocol), omitting incubation in washing solution 3, and immediately scanned using an Agilent G2505B microarray scanner.

The data presented herein were generated in compliance with the MIAME guidelines, and have been deposited in NCBI's Gene Expression Omnibus. They are accessible through GEO Series accession number GSE28499. Raw intensity data were normalized with a non-linear Lowess regression [63]. Differentially expressed genes were identified by an ANOVA fixed effects model [64]. Adjusted *p*-values were calculated by the Benjamini-Hochberg method to control the false-discovery-rate [65]. The list of regulated genes were functionally analysed by identifying common annotation terms and subsequent clustering into biological processes using the DAVID bioinformatics resources as described before [66].

### Subcellular fractionation of macrophages

Cytosolic and nuclear fractions of macrophages were prepared as described before [67]. To this end, equal numbers of primary BMMΦ or RAW264.7 monocytic cells that had been infected with *T. gondii* for 24 hours, or left non-infected and/or stimulated with 300 U/ml IFN-γ during the final 30 min were washed in PBS containing 12.5 mM NaF and 1 mM Na_3_VO_4_, and collected by centrifugation. After having been incubated for 15 min at 4°C in hypotonic lysis buffer (10 mM HEPES, pH 7.8, 10 mM KCl, 2 mM MgCl_2_, 1 mM DTT, 0.1 mM EDTA, 0.1 mM PMSF, 0.1 mM Na_3_VO_4_), cells were disrupted by adding 0.5% Nonidet P-40, vigorous mixing and passage through a 26G needle. Complete cell lysis was assured microscopically after trypan blue staining. After centrifugation at 10,000 × g and 4°C for 1 min, the supernatants containing cytosolic proteins were collected for further analyses (see below). Nuclei were washed in hypotonic lysis buffer (as above), before being extracted in 50 mM HEPES, 50 mM KCl, 300 mM NaCl, 1 mM DTT, 0.1 mM EDTA, 0.1 mM PMSF, 0.1 mM Na_3_PO_4_ and 10% glycerol for 20 min at 4°C. Soluble nuclear proteins were collected after centrifugation.

### Electromobility shift assay (EMSA)

The STAT1-binding activity of nuclear extracts from *Toxoplasma*-infected and non-infected BMMΦ was assayed against the GAS sequence of the Fcγ receptor I (*FcγRI*) gene [68]. Briefly, after annealing 1 µM each of the oligonucleotides (5′-GTATTTCCCAGAA-3′; 5′-CTTTTCTGGGAA-3′), the DNA probe was radiolabeled for 30 min with [^32^P]dATP using Exo(-) Klenow enzyme as recommended (Stratagene, La Jolla, CA, USA), isolated by ammonium acetate/ethanol precipitation and washed. Equal volumes of nuclear extract were then incubated for 45 min with 2 µl of radiolabeled DNA probe [50,000 cpm/µl], 1 µg poly(I)-poly(C), 20 mM HEPES, 50 mM KCl, 4% Ficoll, and 1 mM each DTT and EDTA. Supershift assays were performed by adding of 2 µg of rabbit anti-STAT1α (M-23), rabbit anti-STAT2 (L-20), rabbit anti-p48 (C-20; all supershift grade antibodies from Santa Cruz Biotechnology, Santa Cruz, CA) or normal rabbit IgG. The protein-DNA complexes were resolved using a native 4% polyacrylamide gel in 0.25x TBE and detected by autoradiography.

### Pull down assay of GAS-binding protein complexes

The binding capacity of proteins from *Toxoplasma*-infected and non-infected macrophages towards a GAS-containing DNA oligonucleotide was compared using an in vitro pull-down assay. RAW264.7 cells infected or not with *T. gondii* and stimulated with 300 U/ml IFN-γ for 30 min or left non-stimulated were lysed in 1% Triton X-100, 150 mM NaCl, 50 mM Tris/HCl pH 8.0, 50 mM NaF, 5 mM sodium pyrophosphate, 1 mM PMSF, 1 mM EDTA, 1 mM Na_3_VO_4_ and 5 µg/ml each of leupeptin, aprotinin and pepstatin. After centrifugation, soluble proteins were incubated for 2 hours at 4°C with 1 µM of a biotin-conjugated double-stranded DNA probe containing the GAS sequence of *FcγRI* (for: 5′-biotin-GATGTATTTCCCAGAAAAG-3′; rev: 5′-CTTTTCTGGGAAATACATC-3′) [68]. Thereafter, DNA-protein complexes were incubated for 60 min at 4°C with 30 µl of 50% slurry of UltraLink immobilized streptavidin (Pierce, Rockford, IL, USA). After washing twice in lysis buffer (see above) supplemented with 100 mM KCl and once in lysis buffer, DNA-protein complexes were resolved by one- or two-dimensional PAGE.

### Two-dimensional electrophoresis and mass spectrometry

Proteins isolated by GAS oligonucleotide pull-down assay were desalted and concentrated using Centricon microseperators (MWCO 3,000; Amicon, Beverly, MA). After lyophilization, they were dissolved in 8 M urea, 2% CHAPS, 0.5% IPG buffer, 2.8 mg/ml DTT and bromphenolblue and separated in pH 3–10 Immobiline DryStrips using a IPGphor isoelectric focusing unit as recommended (GE Healthcare, Freiburg, Germany). Immobiline strips were then equilibrated for 15 min in 50 mM Tris/HCl, pH 8.8, 6 M urea, 30% glycerol, 2% SDS, 10 mg/ml DTT and bromphenolblue, loaded on a 10% SDS-polyacrylamide gel and covered with 0.5% agarose. Proteins were separated by standard SDS-PAGE, and visualized by silver staining or colloidal coomassie staining.

Following colloidal Coomassie Brilliant Blue G-250 staining, proteins that had been differentially GAS-precipitated from extracts of *Toxoplasma*-infected macrophages and non-infected controls were analysed by mass spectrometry (MS) essentially as described before [69]. To this end, protein spots were manually excised, washed and destained using alternately 50% acetonitrile (ACN) and 100 mM ammonium bicarbonate 3 times for 5 min. After dehydrating with ACN and drying, protein spots were digested overnight at 37°C with 10 µg/ml trypsin. Peptide samples were extracted with ACN and trifluoroacetic acid and were then co-crystallized with matrix (α-cyano-4-hydroxycinnamic acid) on a stainless steel target using 1 µl matrix and 1 µl sample. Peptide mass maps were generated on an Applied Biosystems Voyager-DE STR time-of-flight mass spectrometer that was operated in delayed reflector mode with an accelerated voltage of 20 kV. Mass spectra were obtained by averaging 50 individual laser shots. Samples were externally calibrated with a peptide mix of des-Arg-bradykinin ([M+H]^+^ 904.46), angiotensin I ([M+H]^+^ 1296.68), Glu1-fibrinopeptide B ([M+H]^+^ 1570.67), ACTH (1-17) ([M+H]^+^ 2093.08), ACTH (18-39) ([M+H]^+^ 2465.19) and the resulting mass spectra were internally calibrated with trypsin autolysis products (*m/z* 842.50 and *m/z* 2211.10). Monoisotopic peptide masses were used for searches against the MSDB or NCBI nr database using the Mascot peptide mass fingerprint software (Matrix Science, Oxford, UK; http://www.matrixscience.com/search_form_select.html) [70]. Carboxamidomethylation and methionine oxidation were considered as variable modifications. Each hit was inspected visually in order to fulfil the following quality control criteria: optimized mass accuracy (approx. 50 ppm), minimal mass deviation (in the mDa range), maximized sequence coverage, highest possible probability score, and maximal number of intense ion signals assigned to the identified protein.

Mass finger print analyses were confirmed by peptide sequence analysis as described before [69]. Data acquisition was performed using MassLynx (v 4.0) software while data were further processed on Protein-Lynx-Global-Server (v 2.1), (Micromass, Manchester, UK). Raw data files were deconvoluted and deisotoped using Max Ent lite algorithm. Processed data were searched against MSDB and Swisspro databases through Mascot search engine using a peptide mass tolerance of 50 ppm and fragment tolerance of 100 mmu.

### SDS-PAGE and immunoblotting

Infected and non-infected macrophages were lysed for 60 min at 4°C in 1% Triton X-100, 150 mM NaCl, 50 mM Tris/HCl pH 8.0, 50 mM NaF, 5 mM sodium pyrophosphate, 1 mM PMSF, 1 mM EDTA, 1 mM Na_3_VO_4_ and 5 µg/ml each of leupeptin, aprotinin and pepstatin. Soluble proteins from complete cellular extracts, from cytosolic and nuclear fractions or after oligonucleotide pull down (see above) were separated by standard SDS-PAGE and were transferred to nitrocellulose by semidry blotting. Non-specific binding sites were blocked using 5% dry skimmed milk, 0.2% Tween-20, 0.02% NaN_3_ in PBS, pH 7.4. Membranes were incubated overnight at 4°C with rabbit anti-STAT1α (M-23; Santa Cruz Biotechnology), mouse monoclonal anti-β-actin (clone AC15; Sigma-Aldrich, Taufkirchen, Germany) or rabbit anti-BRG-1 (H-88; Santa Cruz Biotechnology) diluted in 5% dry skimmed milk, 0.05% Tween-20 in PBS, pH 7.4. Fractionation into nuclear and cytosolic extracts was controlled by incubating membranes with rabbit anti-BRG-1 and rabbit anti-GAPDH (Sigma-Aldrich). Immune complexes were labelled with horseradish-peroxidase-conjugated secondary antibodies (Dianova, Hamburg, Germany) and visualized by ECL chemiluminescence (GE Healthcare, Freiburg, Germany).

### Immunofluorescence staining and confocal microscopy

Parasite development in host cells treated or not with HDAC inhibitors was controlled by immunofluorescence microscopy. Briefly, cells were fixed with 4% paraformaldehyde in 0.1 cacodylate buffer, pH 7.4 for 1 hour and quenched for 10 min in 50 mM NH_4_Cl in PBS. After having been permeabilized for 1 hour using 0.1 mg/ml saponin and 1% BSA in PBS, cells were incubated for 1 hour each with rabbit anti-*Toxoplasma* hyperimmune serum and Cy2-conjugated F(ab')_2_ fragment donkey anti-rabbit IgG (Dianova). The total cell population was stained with 5 µg/ml propidium iodide in PBS. Cells were examined by confocal laser scanning microscopy using Leica TCS SP2.

### FACS analysis

Expression of the MHC class II molecules H2-A/E in macrophages treated or not with HDAC inhibitors was quantified by FACS analysis as described previously [19]. Briefly, RAW264.7 macrophages infected or not with *T. gondii* for 40 hours (parasite-host cell ratio of 1∶1 (RH-LDM) or 3∶1 (NTE, PTG), and treated or not with HDAC inhibitors 2 hour prior to IFN-γ treatment, were collected by scraping and washed twice. Cells (5×10^5^ per staining) were incubated in FACS buffer (1% BSA, 0.1% NaN_3_ in PBS) supplemented with 1 mg/ml normal mouse IgG. They were then consecutively immunolabelled with 2 µg/ml of rat mAb anti-H2-A/E (clone M5/114.15.2. ATCC, Rockville, MD, USA) or isotype control antibody (R35-38, BD Biosciences, Heidelberg, Germany) and R-PE-conjugated donkey F(ab')_2_ fragment anti-rat IgG secondary antibody. After having been washed and fixed in 1% paraformaldehyde, 10.000 cells per sample were analyzed on a FACSCalibur flow cytometer (BD Biosciences).

### Chromatin immunoprecipitation (ChIP)

Acetylation of histones and recruitment of BRG-1 to chromatin was analysed using the ChIP assay kit (Upstate Cell Signaling Solutions, Lake Placid, NY, USA). To this end, *T. gondii*-infected and non-infected control RAW264.7 macrophages stimulated or not with 300 U/ml IFN-γ were fixed in 1% formaldehyde at 37°C for 10 min. After having been washed twice with ice-cold PBS supplemented with 1 mM PMSF and 1 µg/ml each of aprotinin and pepstatin, cells were collected by scraping. Cells (0.75×10^7^ per sample) were then lysed in 0.75 ml of SDS lysis buffer (Upstate) containing 1% SDS, 10 mM EDTA, 50 mM Tris-HCl, pH 8.1, and protease inhibitors (as above) for 10 min on ice. Chromatin was sheared on ice by sonification (Branson Sonifier 250; Branson Ultrasonics, Danbury, CT) with twenty 10 sec-pulses to obtain DNA fragments of 200–800 bp. After centrifugation at 18.000 × g and 4°C for 10 min, 200 µl supernatant were diluted 10-fold in ChIP Dilution buffer (Upstate) containing 0.01% SDS, 1.1% Triton X-100, 1.2 mM EDTA, 16.7 mM Tris-HCl (pH 8.1), 167 mM NaCl and protease inhibitors. In order to quantify the amount of input DNA, 20 µl of diluted DNA from each sample were removed and further processed as described below. The remaining diluted DNA was pre-cleared for 30 min at 4°C with 75 µl of salmon sperm DNA/protein A agarose (Upstate). After centrifugation, the supernatants were incubated overnight at 4°C with 5 µl of ChIP grade rabbit polyclonal anti-acetyl-H4 or anti-acetyl-H3 (both from Upstate), or with 3 µg of ChIP grade anti-BRG-1 (Santa Cruz Biotechnology). A negative control precipitation without antibody was run in parallel. Immune complexes were collected using 60 µl of salmon sperm DNA/protein A agarose for 60 min at 4°C and subsequent centrifugation. Samples were then washed consecutively in low salt wash buffer (0.1% SDS, 1% Triton X-100, 2 mM EDTA, 20 mM Tris-HCl (pH 8.1), 150 mM NaCl), high salt wash buffer (0.1% SDS, 1% Triton X-100, 2 mM EDTA, 20 mM Tris-HCl (pH 8.1), 500 mM NaCl), LiCl wash buffer (0.25 M LiCl, 1% IGEPAL-CA630, 1% sodium deoxycholate, 1 mM EDTA, 10 mM Tris-HCl (pH 8.1)), and twice in TE buffer (10 mM Tris-HCl, 1 mM EDTA, pH 8.0). After the final centrifugation, pellets were incubated twice for 15 min at room temperature in 1% SDS, 0.1 M NaHCO_3_. DNA-protein cross-links of the eluted histone-DNA complexes and of the input DNA (see above) were reversed by treatment for 4 hours at 65°C with 200 mM NaCl, and proteins were digested with 40 µg/ml proteinase K in 10 mM EDTA, 40 mM Tris-HCl, pH 6.5 for 60 min at 45°C. DNA was finally isolated using the PCR purification kit (Qiagen) and was quantified by real-time PCR. In some experiments, the ChIP-IT Express Enzymatic Kit (Active Motif, Carlsbad, CA) was used as recommended by the manufacturer.

### Quantitative real-time PCR

Quantitative real-time PCR of mouse transcripts was used to validate microarray results. Transcripts were selected to represent diverse expression patterns after stimulation of non-infected macrophages with IFN-γ as judged by cDNA microarray. Total RNA from BMMΦ stimulated or not with IFN-γ and/or infected with *T. gondii* as described above was isolated using the RNeasy kit (Qiagen). Following reverse transcription of mRNA using the Omniscript kit (Qiagen), serial dilutions of cDNA were amplified by PCR in a LightCycler with the SYBR Green FastStart DNA Master^Plus^ Set (Roche, Mannheim, Germany) using mouse-specific primer pairs as specified in Table S4. Data were analyzed as described previously [71]. Briefly, the relative gene expression levels were calculated as the fold change between unstimulated (control) and either IFN-γ-stimulated, non-infected BMMΦ or IFN-γ-treated and *T. gondii*-infected BMMΦ (samples) using the formula Ratio  =  (E_target_)^ΔCPtarget(control-sample)^/(E_ref_)^ΔCPref(control-sample)^, where the reference gene was β-actin and the target genes were IFN-γ-regulated genes.

Real-time PCR was also used to quantify DNA isolated by ChIP. To this end, input DNA or ChIP DNA was amplified using primers specific for the CIITA promoter IV (for: 5′-TGCCTTTGGCCCAAAGCTGAA-3′; rev: 5′-TTCTGAGTGCTGCCTGCATGC-3′), H2-Eβ (for: 5′-AAACAACCCAAAGCAAAACC-3′; rev: 5′-TCAGCATCAAAGGAGTCCAG-3′), GBP2 (for: 5′-GCTGGCAACTTCACAAAACA-3′; rev: 5′-TGCCAGAGAACTTGTGAG-GA-3′) or β-actin (for: 5′-GATGACCCAGATCATGTTTGAGAC-3′; rev: 5′-TGCTCG-AAGTCTAGAGCAACATAG-3′). The IFN-γ-induced acetylation of histones or BRG-1 recruitment was calculated according to Ratio  =  (E_ChIP_)^ΔCPChIP(unstimulated – IFN-γ-treated)^/(E_input_)^ΔCPinput(unstimulated – IFN-γ-treated)^, where input and ChIP refer to samples before and after immunoprecipitation, respectively.

### Semi-quantitative RT-PCR

Total RNA was isolated from infected and non-infected RAW264.7 monocytes/macrophages that had been treated with IFN-γ in the absence or presence of HDAC inhibitors. After reverse transcription, H2-Aβ and CIITA cDNAs were amplified as described previously [19]. Amplification of β-actin was used as an internal standard in order to normalize the amount of cDNA present in each sample. PCR products were separated by agarose gel electrophoresis and semi-quantitatively analysed using a BioDoc II digital imaging system (Biometra, Göttingen, Germany).

### Statistical analysis

Results are expressed as means ± S.E.M. of three independent experiments unless otherwise stated. Significant differences between mean values were identified by the Student's *t*-test. *P*-values of less than 0.05 were considered significant.

### Accession numbers

beta-actin (syn. actin, cytoplasmic 1), NM_007393

BRG-1, NM_001174078

CD14, NM_009841

CD69, NM_001033122

CFB (complement factor B), NM_001142706

CIITA (class II transactivator), NM_007575

CXCL11, NM_019494

Fc-gamma receptor 1, NM_010186

FLT1 (FMS-like tyrosine kinase 1), NM_010228

Fyn kinase, NM_008054

GAPDH, NM_008084

gamma-actin (syn. actin, cytoplasmic 2), NM_009609

GBP2 (guanylate binding protein), NM_010260

G-protein linked C3G, NM_054050

H2-Aa, NM_010378

H2-Ab, NM_207105

H2-DMb1, NM_010387

H2-Ea, NM_010381

H2-Eb, NM_010382

histone H3, NM_145073

histone H4, NM_175652

IFIT2 (interferon-induced protein with tetratricopeptide repeats 2), NM_008332

IL-10, NM_010548

iNOS, NM_010927

interferon-gamma (IFN-g), NM_008337

IFN-gamma receptor (IFN-gR1), NM_010511

IGTP (interferon-gamma-induced GTPase), NM_018738

IRF-1 (interferon-regulatory factor 1), NM_008390

IP-10 (syn. CXCL10), NM_021274

MIG (syn. CXCL9), NM_008599

MMP9, NM_013599

MX1 (myxovirus (influenza virus) resistance 1), NM_010846

myosin 1, NM_001080775

p48 (syn. IRF9, ISGF3γ), NM_001159417

PI3K (catalytic alpha chain), NM_008839

Rap-1, NM_145541

SHP-1 (syn. PTPN6), NM_013545

SHP-2 (syn. PTPN11), NM_011202

STAT1 (signal transducer and activator of transcription 1), NM_009283

STAT2 (signal transducer and activator of transcription 2), NM_019963

TNF-alpha, NM_013693

tropomyosin 3, NM_022314

## Supporting Information

Figure S1 Validation of microarray data by quantitative RT-PCR. (A) Transcripts that were regulated or not by IFN-γ in primary BMMΦ were randomly selected from the microarray. The change of mRNA abundance following *T. gondii* infection (x-fold) as measured by microarray was compared with the respective real-time PCR data. (B) Microarray data on the IFN-γ-regulated mRNA change as observed after *Toxoplasma* infection were plotted against the corresponding data retrieved from real-time PCR. The correlation coefficient r was obtained after a linear regression analysis.
(TIF)

Figure S2 HDAC inhibitor MS-275 restores defective H2-A/E expression in parasite-positive and parasite-negative macrophages following infection with *T. gondii*. RAW264.7 macrophages were infected with GFP-expressing type II PTG and type I RH parasites at parasite-host cell ratios of 3∶1 or 1∶1, respectively, or were left non-infected. Cells were treated with 2 µM MS-275, or were mock treated as indicated, and two hours later, were stimulated or not with IFN-γ. Forty hours after infection, cells were immunolabelled with anti-H2-A/E, and were analysed by flow cytometry. Results are from a representative experiment out of two. Percentages of cells within the individual quadrants or within the two upper and lower quadrants are indicated.
(TIF)

Figure S3 Replication of *T. gondii* in the presence of HDAC inhibitors. (A) RAW264.7 macrophages were infected with *T. gondii* at a parasite-host cell ratio of 3∶1 and were treated at 1 hour post infection with MS-275, sodium butyrate, MC1568 (all at 2 µM), or nicotinamide (5 mM), or were mock treated. Fourty hours after infection, cells were fixed and labelled with polyclonal anti-*T. gondii* serum and propidium iodide. Representative images obtained by confocal fluorescence microscopy are shown. (B) After treatment of *T. gondii* with HDAC inhibitors as described in (A), the average number of parasites per parasitophorous vacuole was calculated. Results are means ± S.E.M. from two experiments.
(TIF)

Figure S4 Defective IFN-γ-induced H2-A/E expression after infection of murine macrophages with *T. gondii* is restored by HDAC inhibitors with different substrate specificities. (A) RAW264.7 macrophages were infected with *T. gondii* (parasite-host cell ratio 3∶1, lower panel) or were left non-infected (upper panel), and were stimulated or not with IFN-γ as indicated. Two hours prior to cytokine stimulation, cells were treated with MS-275, nicotinamide, or MC1568, or were mock treated. Forty hours after infection, cells were immunolabelled with anti-H2-A/E, and were analysed by flow cytometry. Results are from a representative experiment out of three, data are the percentages of cells within the individual quadrants. (B) Murine macrophages were infected with *T. gondii* and/or treated with IFN-γ and HDAC inhibitors as described in (A). Cells were immunolabelled with anti-mouse H2-A/E or an isotype-matched control antibody, and were analysed by flow cytometry. Data represent the mean percentages ± S.E.M. of positive cells from three independent experiments. *: *p*<0.05, ns: not significant (Student's *t*-test).
(TIF)

Table S1 Expression of IFN-γ-inducible genes in *T. gondii*-infected and non-infected BMMΦ. Differentially expressed genes after treatment of non-infected BMMΦ with IFN-γ (IFN-γ, n.i. vs untreated, n.i.) were identified by ANOVA; those at least four-fold up-regulated (absolute value) at a false-discovery rate ≤ 1% were included in the list. Expression in non-infected BMMΦ was compared with that in *Toxoplasma*-infected BMMΦ (IFN-γ, *T. gondii* vs untreated, *T. gondii*) and the parasite effect was calculated [x-fold].
(XLSX)

Table S2 Expression of IFN-γ-repressed genes in *T. gondii*-infected and non-infected BMMΦ. Differentially expressed genes after treatment of non-infected BMMΦ with IFN-γ (IFN-γ, n.i. vs untreated, n.i.) were identified by ANOVA; those at least four-fold down-regulated (absolute value) at a false-discovery rate ≤ 1% were included in the list. Expression in non-infected BMMΦ was compared with that in *Toxoplasma*-infected BMMΦ (IFN-γ, *T. gondii* vs untreated, *T. gondii*) and the parasite effect was calculated [x-fold].
(XLSX)

Table S3 IFN-γ-regulated biological processes which are predominantly counteracted by *T. gondii*. Genes that were differentially expressed after treatment of non-infected BMM with IFN-γ (at least four-fold; identified by ANOVA), but not in those previously infected with *T. gondii* were subjected to the functional annotation of biological processes using the DAVID Bioinformatics resource. Major functional targets of parasite interference were identified by including those GO annotation terms that were common to at least five genes, and that were significantly enriched (modified Fisher's exact test, P<0.1). The level of enrichment of genes was determined against all *Mus musculus* genes.
(XLSX)

Table S4 Primers used for validation of microarray results by quantitative real-time PCR.
(DOC)
