# Supplementary figures and images for: Impaired Chromatin Remodelling at STAT1-Regulated Promoters Leads to Global Unresponsiveness of Toxoplasma gondii-Infected Macrophages to IFN-γ
Source: PLoS Pathog. 2012 Jan 19;8(1):e1002483. doi: 10.1371/journal.ppat.1002483 (PMC3262016; doi:10.1371/journal.ppat.1002483)

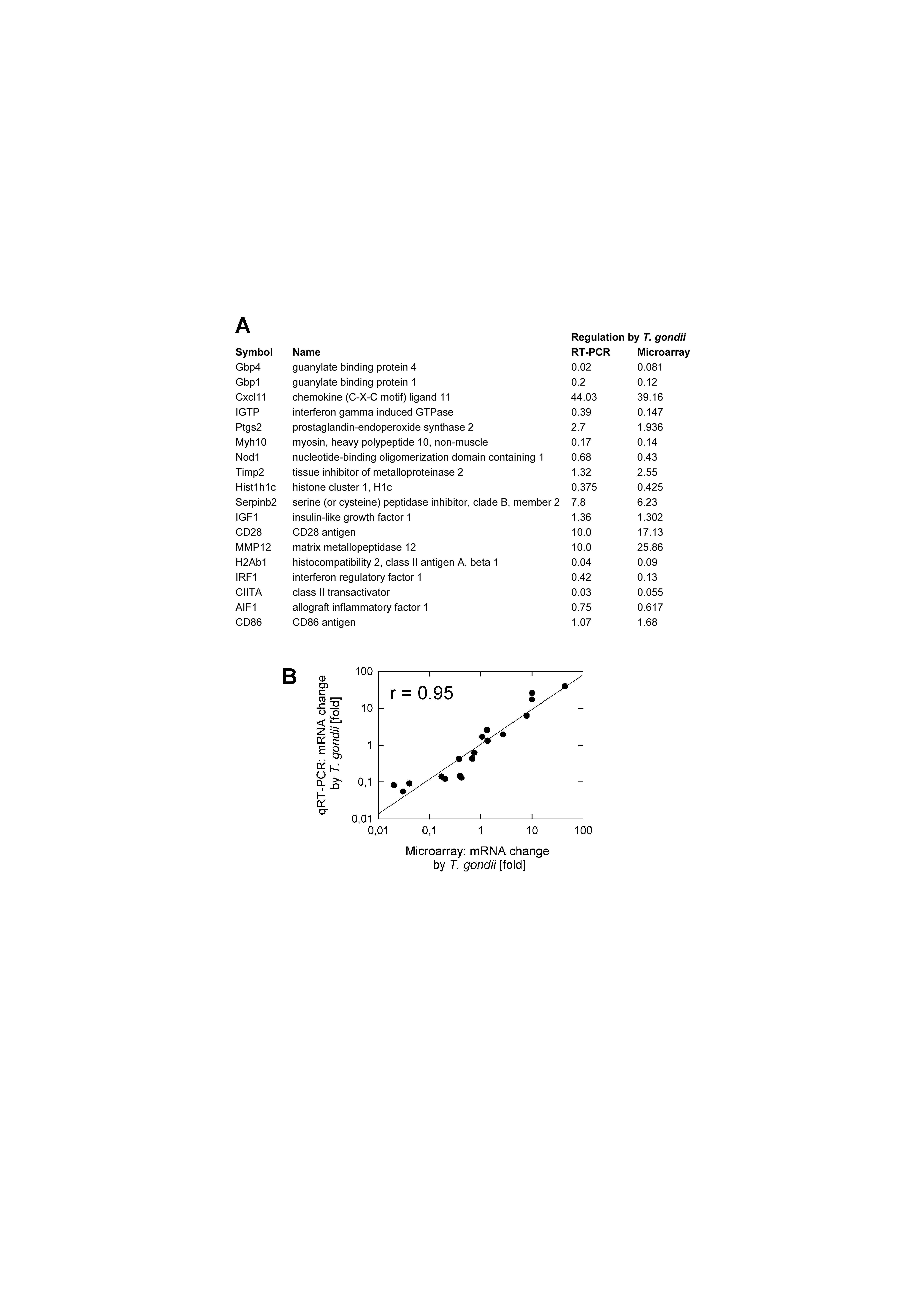

Supplement: Figure S1 — Validation of microarray data by quantitative RT-PCR. (A) Transcripts that were regulated or not by IFN-γ in primary BMMΦ were randomly selected from the microarray. The change of mRNA abundance following T. gondii infection (x-fold) as measured by microarray was compared with the respective real-time PCR data. (B) Microarray data on the IFN-γ-regulated mRNA change as observed after Toxoplasma infection were plotted against the corresponding data retrieved from real-time PCR. The correlation coefficient r was obtained after a linear regression analysis. (TIF) [file ppat.1002483.s001.tif]

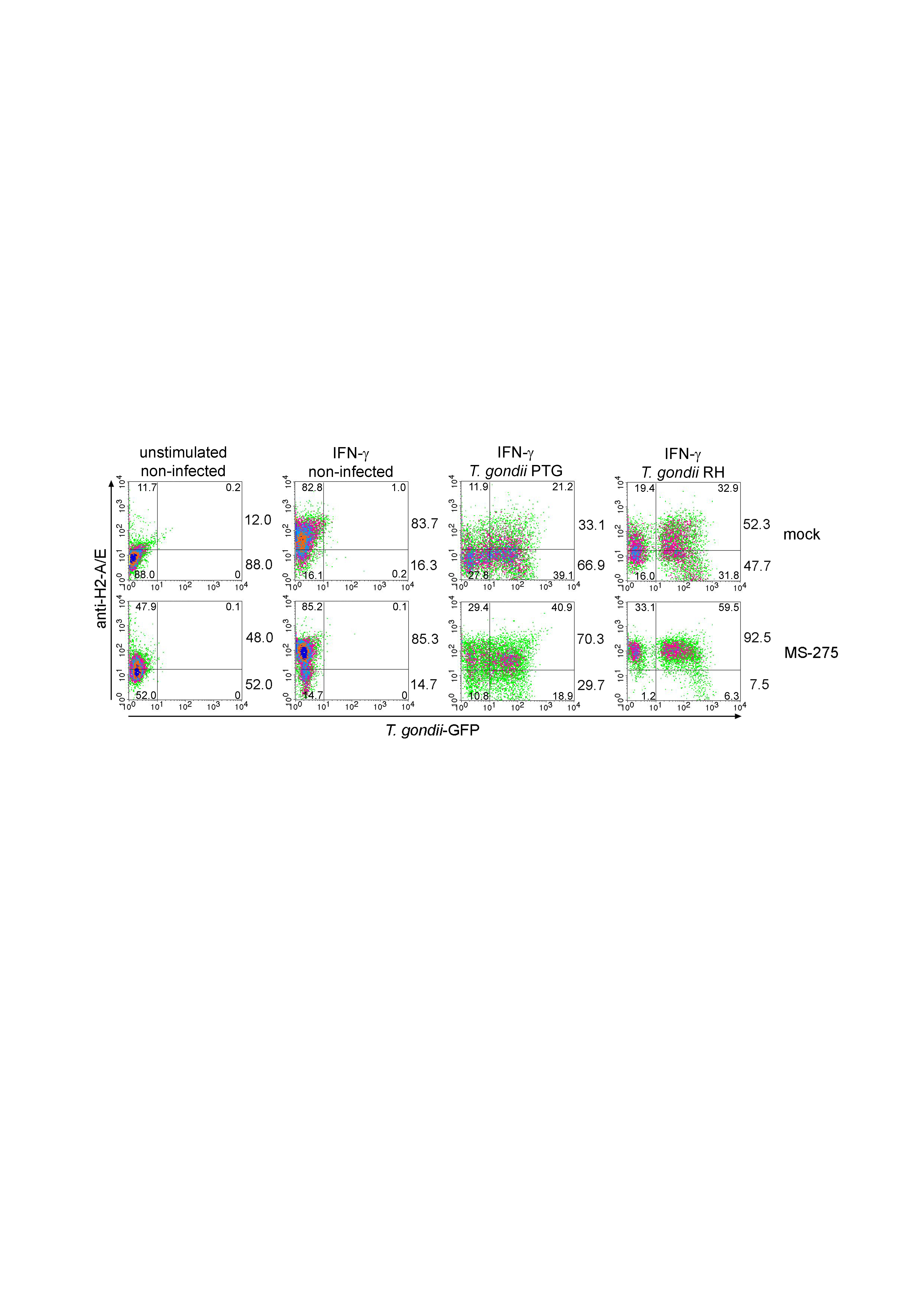

Supplement: Figure S2 — HDAC inhibitor MS-275 restores defective H2-A/E expression in parasite-positive and parasite-negative macrophages following infection with T. gondii. RAW264.7 macrophages were infected with GFP-expressing type II PTG and type I RH parasites at parasite-host cell ratios of 3∶1 or 1∶1, respectively, or were left non-infected. Cells were treated with 2 µM MS-275, or were mock treated as indicated, and two hours later, were stimulated or not with IFN-γ. Forty hours after infection, cells were immunolabelled with anti-H2-A/E, and were analysed by flow cytometry. Results are from a representative experiment out of two. Percentages of cells within the individual quadrants or within the two upper and lower quadrants are indicated. (TIF) [file ppat.1002483.s002.tif]

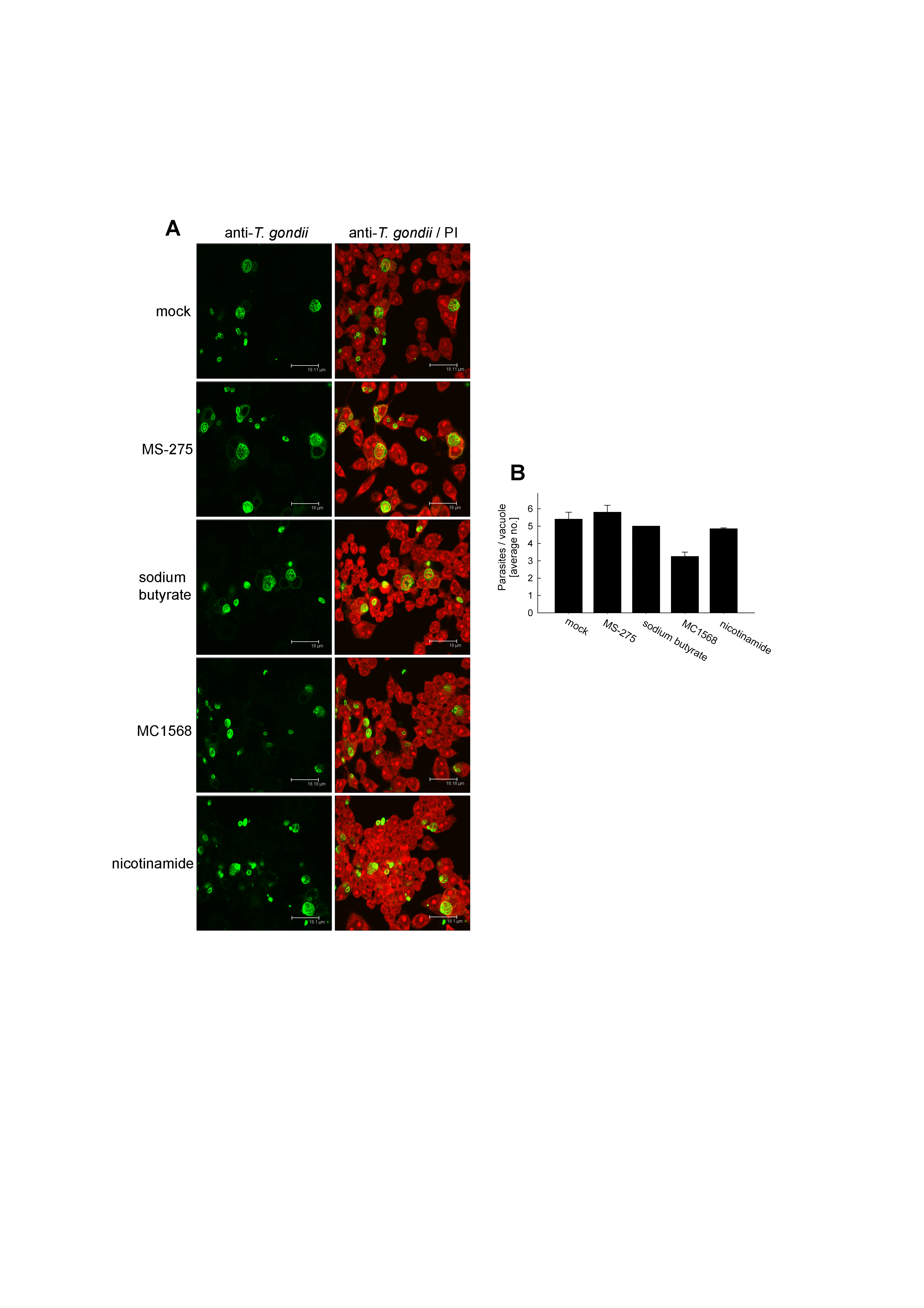

Supplement: Figure S3 — Replication of T. gondii in the presence of HDAC inhibitors. (A) RAW264.7 macrophages were infected with T. gondii at a parasite-host cell ratio of 3∶1 and were treated at 1 hour post infection with MS-275, sodium butyrate, MC1568 (all at 2 µM), or nicotinamide (5 mM), or were mock treated. Fourty hours after infection, cells were fixed and labelled with polyclonal anti-T. gondii serum and propidium iodide. Representative images obtained by confocal fluorescence microscopy are shown. (B) After treatment of T. gondii with HDAC inhibitors as described in (A), the average number of parasites per parasitophorous vacuole was calculated. Results are means ± S.E.M. from two experiments. (TIF) [file ppat.1002483.s003.tif]

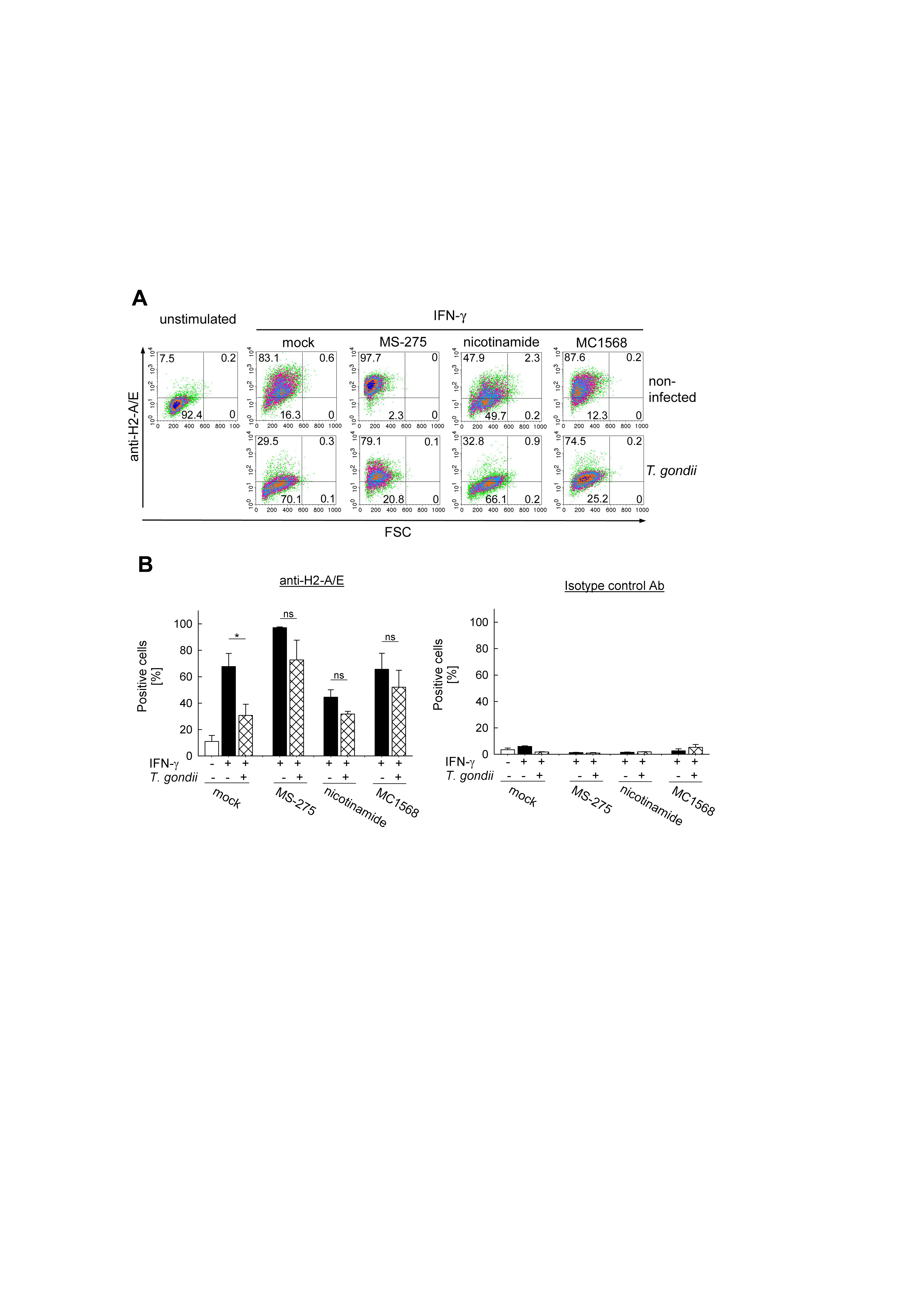

Supplement: Figure S4 — Defective IFN-γ-induced H2-A/E expression after infection of murine macrophages with T. gondii is restored by HDAC inhibitors with different substrate specificities. (A) RAW264.7 macrophages were infected with T. gondii (parasite-host cell ratio 3∶1, lower panel) or were left non-infected (upper panel), and were stimulated or not with IFN-γ as indicated. Two hours prior to cytokine stimulation, cells were treated with MS-275, nicotinamide, or MC1568, or were mock treated. Forty hours after infection, cells were immunolabelled with anti-H2-A/E, and were analysed by flow cytometry. Results are from a representative experiment out of three, data are the percentages of cells within the individual quadrants. (B) Murine macrophages were infected with T. gondii and/or treated with IFN-γ and HDAC inhibitors as described in (A). Cells were immunolabelled with anti-mouse H2-A/E or an isotype-matched control antibody, and were analysed by flow cytometry. Data represent the mean percentages ± S.E.M. of positive cells from three independent experiments. *: p<0.05, ns: not significant (Student's t-test). (TIF) [file ppat.1002483.s004.tif]
