# Supplementary material for: Impaired Chromatin Remodelling at STAT1-Regulated Promoters Leads to Global Unresponsiveness of Toxoplasma gondii-Infected Macrophages to IFN-γ
Source: PLoS Pathog. 2012 Jan 19;8(1):e1002483. doi: 10.1371/journal.ppat.1002483 (PMC3262016; doi:10.1371/journal.ppat.1002483)
Supplement: Table S4 — Primers used for validation of microarray results by quantitative real-time PCR. (DOC) [file ppat.1002483.s008.doc]

**Table S4:** Primers used for validation of microarray results by quantitative RT-PCR

| mRNA | Accession No. | Forward primer | Reverse primer |
| --- | --- | --- | --- |
| β-actin | M12481 | 5’-GATGACCCAGATCATGTTTGAGAC-3’ | 5’-TGCTCGAAGTCTAGAGCAACATAG-3’ |
| Gbp4 | NM_008620 | 5’-GGATAGACTGTGATACACAC-3’ | 5’-GTCCTCATAGGAGTACAGAA-3’ |
| Gbp1 | NM_010259 | 5’-TCATGCTATCTGCAGACATTG-3’ | 5’-TCTCCATTCTGTGTAGAAGTC-3’ |
| Cxcl11 | NM_019494 | 5’-GGCTTCCTTATGTTCAAACAG-3’ | 5’-GTTCCTGGCACAGAGTTCTTA-3’ |
| IGTP | NM_018738 | 5’-TGAACAAGTTCCTCAGGGTTC-3’ | 5’-TGAAGGAACGGGCTATTACAG-3’ |
| Ptgs2 | NM_011198 | 5’-ACCTCTCTGAACTATGGTGTG-3’ | 5’-CTTGATGCCCGAATTCTAATG-3’ |
| Myh10 | NM_175260 | 5’-CAAATTCAGCCACTGCTTTGA-3’ | 5’-GGTACATGATATGAAGCACAG-3’ |
| Nod1 | NM_172729 | 5’-GCTCATCACAGACTTTGGTTC-3’ | 5’-ATCTGCAACATTCCAACTGTC-3’ |
| Timp2 | NM_011594 | 5’-AACCCTGTCCTAACCATCAGA-3’ | 5’-TGCTACGAAAGGCATCAACAC-3’ |
| Hist1h1c | NM_015786 | 5’-TTGCACAGTTAAGTTGGACTC-3’ | 5’-AGCATTACCAAGAAACCTCAG-3’ |
| Serpinb2 | NM_011111 | 5’-CAGTTATGACAGGAAGAACTG-3’ | 5’-AAGCACTGAAGACTGCTATAC-3’ |
| IGF1 | NM_001111274 | 5’-ATGCTCTTCAGTTCGTGTGTG-3’ | 5’-TTCCTGCACTTCCTCTACTTG-3’ |
| CD28 | NM_007642 | 5’-CATTTAGTCAACGGAGTGAG-3’ | 5’-CATAAACATAGCAGTGCAAG-3’ |
| MMP12 | NM_008605 | 5’-CTTTCTGTCACCAAAGCTTGAG-3’ | 5’-CAACCTTCTTCACAGATGCAG-3’ |
| H2Ab1 | NM_207105 | 5’-CAGTTCAAGGGCGAGTGCTAC-3’ | 5’-CAGAGTGTTGTGGTGGTTGAG-3’ |
| IRF1 | NM_008390 | 5’-GGTTCCCAAGCCAGTGAAATG-3’ | 5’-TAGTCAAGAGTCACGCCAAGG-3’ |
| CIITA | NM_007575 | 5’-GTGAGCGCTGGTAGACAGAAC-3’ | 5’-GCCTTTTGGTTCACACCCAGG-3’ |
| AIF1 | NM_019467 | 5’-ACCCACCTAGAGCTGAAGAGA-3’ | 5’-TTAGTCTGACTCTGGCTCACG-3’ |
| CD86 | NM_019388 | 5’-TGTGCAAGGATAAGACAGCCA-3’ | 5’-TCCTTCCACACAAGCCATGAG-3’ |
